# SARS-CoV-2-Specific IgG Levels Are Inversely Associated with ACPA-Reactive Pathological and Natural Autoantibody Profiles in Residual Diagnostic Samples

**DOI:** 10.3390/vaccines14100888

**Published:** 2026-10-10

**Authors:** Alain Le Roux, Nancy Zeineddine, Katalin Böröcz, Dávid Szinger, Diana Simon, Ferenc Boldizsár, Szabina Erdo-Bonyar, Péter Németh, Tímea Berki

**Affiliations:** Department of Immunology and Biotechnology, Clinical Centre, Medical School, University of Pécs, 7624 Pécs, Hungary

**Keywords:** SARD, rheumatoid arthritis, SARS-CoV-2, autoantibodies, natural autoantibodies, ACPA, rheumatoid factor, humoral immunity, heat-shock proteins

## Abstract

**Background/Objectives:** SARS-CoV-2-specific antibody levels vary among patients with systemic autoimmune rheumatic diseases (SARDs). In rheumatoid arthritis (RA), the most prevalent SARD, this is further shaped by rheumatoid factors (RFs). Attenuated post-vaccination antibody levels in RA are documented, but links to broader autoreactivity remain underexplored. We therefore examined pathological (pAAbs) and natural autoantibodies (nAAbs) in relation to SARS-CoV-2-specific IgG. **Methods:** A decade-long dataset (2014–2024; 73,747 patients) gave descriptive context, not the substudy population. SARS-CoV-2 IgG and nAAbs were evaluated in 200 residual specimens collected between November 2023 and February 2024. SARS-CoV-2-specific IgG was interpreted as an antiviral readout, not a protection correlate (clinical/exposure data unavailable). QuantiVac (wild-type) and Omicron BA.1 IgG were compared across ACPA-reactive (n = 44/38), anti-SSA/SSB-dominant (n = 24/22), anti-dsDNA-reactive (n = 33/32), aPL AAb-dominant (n = 15/14), and autoantibody-negative groups (n = 70/68). **Results:** ACPA-reactive samples showed lower antiviral IgG across both assays. After age/sex adjustment, comparator groups had 2.58–3.30-fold higher QuantiVac and 2.70–4.87-fold higher Omicron BA.1 IgG. Antiviral IgG correlated inversely with ACPA IgG (ρ to −0.474) and RF isotypes (ρ = −0.299 to −0.400). ACPA-reactive samples showed increased CS-IgM and HSP60/HSP70 IgG/IgM reactivity, which correlated positively with ACPA IgG and RF isotypes. **Conclusions:** In this exploratory analysis, an ACPA-reactive serological profile was associated with lower SARS-CoV-2 IgG and higher, positively correlated CS- and HSP-directed autoantibody levels, suggesting a shared rather than independent basis for these autoreactive compartments. These cross-sectional associations persisted after age/sex adjustment but cannot be separated from unmeasured clinical, treatment, or exposure factors.

## 1. Introduction

It remains unclear whether reduced SARS-CoV-2-specific humoral responsiveness is a general feature of systemic autoimmune rheumatic diseases (SARDs) [1,2,3] or preferentially reflects specific underlying autoimmune serological profiles. In this context, rheumatoid arthritis (RA) provides a particularly informative model by virtue of three converging features: it is the most prevalent SARD worldwide [4]; reduced SARS-CoV-2 vaccine antibody responses have been increasingly reported in RA [2,5,6] and RA-associated serology is characterized by highly prevalent, pleiotropic rheumatoid factors (RFs) [7,8,9,10,11]—antibodies that themselves bind the Fc region of other antibodies and are not specific to RA, which may further complicate post-vaccination humoral responses. Although the impact of immunomodulatory therapies on post-vaccination antibody responses in RA is well established [2,5,6,12,13,14], it remains unclear whether SARS-CoV-2-specific IgG levels are also linked to the broader autoreactive serological milieu, reflecting an interplay between targeted antiviral and autoimmune humoral immunity [1,15,16,17].

We therefore aimed to examine whether SARS-CoV-2-specific IgG levels were associated with RA-related autoreactivity across two complementary compartments: diagnostically relevant pathological autoantibodies (pAAbs, i.e., disease-associated autoantibodies with established diagnostic value) and selected homeostasis-associated natural autoantibody (nAAb) reactivities.

For this purpose, we used pseudonymized residual diagnostic samples and therefore applied a serology-based approach. Linked vaccination and infection histories, as well as clinical outcome data relevant to protective immunity, were unavailable. Accordingly, SARS-CoV-2-specific binding IgG was interpreted as a quantitative antiviral humoral readout rather than as a direct correlate of protection [18,19]. Within this framework, pAAbs and nAAbs were likewise considered serological features of the underlying autoreactive humoral milieu rather than validated clinical predictors.

Within the pAAb compartment, anti-citrullinated protein antibody (ACPA)-reactive RA-associated samples commonly show concomitant RF reactivity [7,8,9,10]. RFs recognize the Fc region of IgG [7,8,10,11] and participate in IgG-containing immune complexes, providing a biological rationale for examining their relationship with antiviral IgG [8,10,11]. In parallel, accumulating evidence links alterations in nAAb profiles to RA-associated autoimmunity [20,21,22,23,24,25]. Although traditionally regarded as relatively conserved and constitutively present components of the immune repertoire [16,26,27,28], nAAbs are increasingly recognized as functional contributors to immune homeostasis, with protective, compensatory, and anti-inflammatory roles [15,29,30,31,32,33]. Their levels and repertoire composition may also change in response to antigenic stimulation and in SARDs [34,35,36,37,38,39].

We first used a decade-long regional autoimmune-diagnostics dataset from Southern Transdanubia, Hungary (2014–2024), to characterize the relative positivity of RA-associated serological markers [4] within our diagnostic setting and to establish the context of the broader diagnostic cohort. Within a smaller cross-sectional sample set derived from this de-duplicated parent cohort, we then examined whether ACPA-reactive samples displayed lower SARS-CoV-2-specific IgG levels than comparator serological profiles and whether antiviral IgG magnitude tracked with the broader autoreactive humoral architecture across pAAb and nAAb compartments.

A distinctive feature of the present study is the integration of these two autoreactive compartments within a diagnostically anchored, serology-centered framework. This approach allowed us to assess whether otherwise distinct pAAb and nAAb reactivities form a coordinated serological pattern associated with antiviral antibody magnitude. The resulting relationships are hypothesis-generating and provide a basis for prospective evaluation of broader autoreactive humoral profiles in relation to antiviral IgG levels. This study is precisely scoped to address one of three related questions in this field: whether clinically confirmed RA is associated with diminished vaccine immunogenicity; whether immunomodulatory therapies alter antibody levels; or whether the magnitude of RA-associated autoantibody reactivity is cross-sectionally associated with SARS-CoV-2-specific IgG. We focus on this third question because, unlike the first two, it can be examined directly within a residual-sample framework without requiring confirmed diagnoses or treatment data; the first two are addressed elsewhere by the clinically characterized cohorts cited above. Within this study, the group-level comparisons of SARS-CoV-2 IgG (Section 3.2 and Section 3.3) constitute the primary, confirmatory analysis, while the correlation and natural-autoantibody analyses (Section 3.4, Section 3.5 and Section 3.6) are exploratory and hypothesis-generating.

## 2. Materials and Methods

### 2.1. Study Design and Setting

The study was performed at the MSZ EN ISO 15189:2023-accredited Autoimmune Routine Diagnostics Laboratory of the Clinical Centre, University of Pécs, and was approved by the Regional Research Ethics Committee (2-PTERKEB2026/1). A decade-long (2014–2024), retrospective regional analysis of SARD-related diagnostic autoantibody positivity was performed in Southern Transdanubia, Hungary, using data from our Autoimmune Routine Diagnostic Laboratory with designated healthcare provision for the regional catchment area (73,747 patients, aged 18–80 years). Patient records were de-duplicated across the entire database (not separately per assay) by sorting all records by patient identifier and retaining the first record per identifier; this yielded the 73,747 unique patients reported above (see Reconciliation of sample sizes, below). For each individual assay, positivity was assigned based on any measurement exceeding the assay-specific cut-off at any time during the 2014–2024 observation period (i.e., patients were classified as ever positive rather than positive at first test); patients with only negative measurements for a given assay were classified as negative for that assay. This ever-positive approach can yield higher positivity estimates than a first-test-only definition and may differ across assays according to how frequently each was repeated in individual patients; this is noted as a limitation. Assay methodology was not necessarily stable across the decade-long observation period: for example, the antinuclear antibody (ANA) screen and extractable nuclear antigen (ENA) screen each used two different reference ranges and manufacturers across two testing eras within 2014–2024 (Supplementary Table S1), and analogous undocumented changes in reagents, instruments, or calibration for other assays cannot be excluded. Because clinical diagnoses, treatment histories, disease activity scores, vaccination histories, and infection histories were unavailable, the serogroups described below should be interpreted throughout as autoantibody-defined serological profiles rather than confirmed clinical disease cohorts.

### 2.2. Diagnostic Autoantibody Positivity in the Regional Cohort (Figure 1)

Assay-specific diagnostic positivity thresholds were retained (Supplementary Table S1), and positivity was assigned on an ever-positive basis (any measurement exceeding the respective cut-off at any time during the observation period; see Study design and setting, above). The analysis included: ANA screen (n = 41,764), RF IgM (n = 24,515), RF IgG (n = 24,391), RF IgA (n = 24,521), anti-dsDNA IgG (n = 27,118), ACPA IgG (n = 26,437), anti-nucleosome IgG (n = 31,590), ENA screen (n = 35,085), aCL/β2-GPI IgM and IgG (n = 30,362 each), anti-prothrombin (a-PT) IgG and IgM (n = 22,619 each), anti-proteinase 3 (PR3) IgG (n = 32,283), and anti-myeloperoxidase (MPO) IgG (n = 32,282). This cohort comprised 128,847 laboratory test episodes (individual blood draws/test orders) from the 73,747 de-duplicated patients described above; because a single test episode frequently included more than one of the 14 assays listed here (e.g., ANA, RF, and anti-dsDNA were often requested together from the same specimen), the number of test episodes is smaller than the total of 405,948 obtained by summing the assay-specific n values above, which counts each assay result separately regardless of how many were ordered together in the same episode. Major pAAb positivity proportions were calculated as the proportion of positive individuals among all individuals (de-duplicated patients) tested for each assay, with 95% confidence intervals (CIs) calculated using the Wilson score method. Because this cohort comprises individuals referred for autoimmune diagnostic testing rather than a general-population sample, these values represent diagnostic test positivity rates within a referred clinical population and should not be interpreted as general-population or disease prevalence.

**Figure 1:**
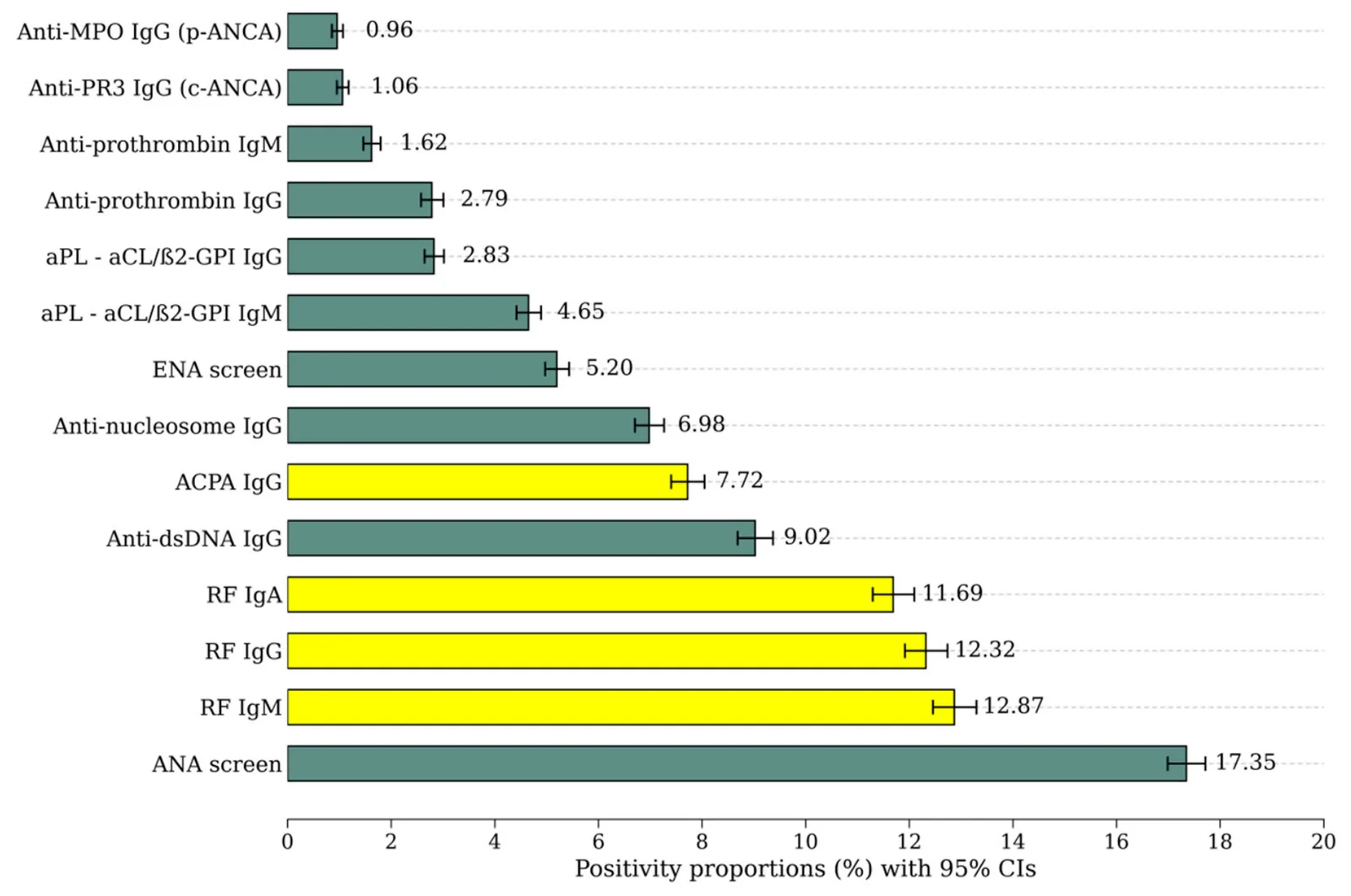
Major pAAb positivity proportions, within this referred diagnostic population, with 95% confidence intervals (CIs). Forest plot showing the positivity rates of routinely assessed pAAbs, with point estimates expressed as percentages and horizontal error bars representing 95% Wilson score confidence intervals (CIs). The number of individuals tested and positive for each marker was: ANA screen, 7245/41,764; RF IgM, 3155/24,515; RF IgG, 3005/24,391; RF IgA, 2866/24,521; anti-dsDNA IgG, 2447/27,118; ACPA IgG, 2041/26,437; anti-nucleosome IgG, 2205/31,590; ENA screen, 1824/35,085; aCL/β2-GPI IgM, 1412/30,362; aCL/β2-GPI IgG, 858/30,362; anti-PT IgG, 630/22,619; anti-PT IgM, 367/22,619; anti-PR3 IgG (c-ANCA), 342/32,283; and anti-MPO IgG (p-ANCA), 309/32,282. RA-associated serological markers (ACPA IgG and RF IgA, IgM and IgG) are highlighted in yellow, while the remaining autoantibody specificities are shown in green. Positivity ranged from 17.35% for ANA screening to 0.96% for anti-MPO IgG. ACPA, anti-citrullinated protein antibody; RF, rheumatoid factor; ANA, antinuclear antibody; dsDNA, double-stranded DNA; ENA, extractable nuclear antigen; aCL, anticardiolipin; β2-GPI, β2-glycoprotein I; PT, prothrombin; PR3, proteinase 3; MPO, myeloperoxidase; ANCA, antineutrophil cytoplasmic antibody.

### 2.3. Serum Antibody Assays

#### 2.3.1. Specimen Collection and Study Populations

Residual serum specimens for the SARS-CoV-2 IgG and nAAb substudy were collected between November 2023 and February 2024, after SARS-CoV-2 vaccines had become widely available in the region; sampling dates did not differ systematically among serological groups. Within this window, all diagnostic specimens with sufficient residual serum volume for the planned testing were included, making specimen selection a volume-based convenience sample rather than a randomized or consecutive one. Specimens were pseudonymised and tracked at the sample level; whether any single patient contributed more than one specimen to this substudy was not additionally verified beyond this specimen-level pseudonymisation, and this possibility cannot be excluded. Unlike the 18–80-year restriction applied to the large-cohort diagnostic-positivity analysis above (Figure 1), no age restriction was applied here, because the substudy was designed to capture SARS-CoV-2 exposure across the full age spectrum rather than a specific autoimmune disease phenotype; consequently, this substudy population spans 5–91 years.

For each specimen, pAAb-based group allocation, SARS-CoV-2 IgG measurement, and nAAb measurement were all performed on aliquots from the same diagnostic sample, ensuring temporal concordance between serological classification and both antibody measurements. Samples for both the SARS-CoV-2 IgG and nAAb assays were processed in study-code order, independent of serological group, with laboratory staff blinded to group allocation throughout.

#### 2.3.2. Relationship to the Large Diagnostic Cohort

The decade-long, 73,747-patient diagnostic cohort described above (Study design and setting) is the source population for the diagnostic-positivity analysis only (Figure 1); it is not the source pool for this substudy, which instead comprises the 200 residual specimens described above, subsequently allocated to serogroups using the algorithm described below (Principles of serological group allocation). The per-marker and per-comparison sample sizes reported in the Results and in Supplementary Tables S1–S7 (typically n < 100) are further reduced from this base of 200, owing to assay-specific missing values; these successive reductions are stated explicitly wherever the corresponding sample size is reported. A STROBE-style flow diagram summarizing the derivation of both populations, including a worked, independently verified example tracing the ACPA (CCP) assay from raw test episodes through to the patient-level counts reported in Supplementary Table S1, is provided in Supplementary Figure S3.

#### 2.3.3. Anti-SARS-CoV-2 IgG (Used for Figure 2, Figure 3 and Figure 4)

Anti-SARS-CoV-2 IgG was quantified using the EUROIMMUN (Euroimmun GmbH, Lübeck, Germany) Anti-SARS-CoV-2 QuantiVac ELISA (IgG) (REF EI 2606-9601-10 G) and EUROIMMUN Anti-SARS-CoV-2 Omicron ELISA (IgG) (REF EI 2606-9601-30 G), according to the manufacturer’s instructions. Both assays use recombinant S1 spike protein expressed in HEK293 cells: the QuantiVac assay is coated with the S1 domain of the Wuhan-Hu-1 strain, the Omicron assay with the S1 domain of the Omicron BA.1 variant. Bound IgG was detected using a peroxidase-labelled anti-human IgG conjugate, with absorbance measured at 450 nm and a reference wavelength of 620–650 nm. Antibody concentrations were calculated from six-point calibration curves and expressed in reference units/mL (RU/mL), with an analytical measurement range of 1–120 RU/mL; samples above this range were re-analysed after dilution. Results were classified as negative <8 RU/mL, borderline 8 to <11 RU/mL, and positive ≥11 RU/mL.

Because rheumatoid factor (RF) was positive in the great majority of ACPA-dominant samples in this cohort, potential RF interference with these assays was specifically considered. Per the manufacturers’ validation data, RF-positive sera were included in the interference panels for both assays: no false-positive reactivity was observed for the QuantiVac ELISA (0/40 RF-positive samples reactive), whereas the Omicron ELISA showed a low rate of false-positive reactivity in RF-positive sera (2/37, 5.4%), characterized by the manufacturer as an isolated finding rather than evidence of general interference. Neither assay’s documentation specifies a dedicated RF-blocking reagent. Given the high RF-positivity rate in the ACPA-dominant group, a small degree of RF-related false-positive reactivity cannot be fully excluded, particularly for the Omicron assay; this is noted as a limitation.

#### 2.3.4. pAAb Panel (Used for Figure 2, Figure 3, Figure 4 and Figure 5)

ACPA IgG, RF isotypes (IgA, IgM, IgG), anti-dsDNA, ANA, ENA, and aCL/β2-GPI were measured using the routine diagnostic assays listed in Supplementary Table S1 (predominantly ORGENTEC by Sebia (Mainz, Germany) kits), each with its own assay-specific cut-off.

#### 2.3.5. Natural Autoantibody (nAAb) Assays (Used for Figure 4 and Figure 5)

Serum IgG and IgM reactivities against citrate synthase (CS), HSP60, and HSP70 were quantified using in-house indirect ELISAs, adapted from previously described methods [15,24], yielding six reactivities. Citrate synthase (porcine heart; Sigma-Merck, Darmstadt, Germany) and recombinant human HSP60 (Abcam, Cambridge, UK) were coated at 1 µg/mL, and recombinant human HSP70 (Abcam) at 1.1 µg/mL; sera were incubated for 50 min (IgG plates) or 45 min (IgM plates). Results were expressed in arbitrary units/mL (AU/mL) based on four-parameter logistic (4-PL) standard curves aligned to measured optical densities (450/620 nm) of an in-house reference pool, using a Siemens BEP 2000 Advance system (Siemens Healthcare Diagnostics Products GmbH, Marburg, Germany). For each antigen, sera were diluted once (1:100) and transferred with identical layout to separate IgG and IgM plates; each plate carried a complete calibrator series and blank wells, and all sera were measured with the same reference pool and reagent lots. Assays were performed according to the internal protocol ML AU NAAB-01 (mean intra-assay CV 6.7–13.3%; inter-assay CV ≤ 30%; limit of detection 0.033–2.22 AU/mL across the six assays; calibrator recovery 78–114%); full assay validation data are available from the corresponding author on reasonable request. Patient identifiers were matched to residual serum specimens retained in the routine diagnostic laboratory archive at −20 °C under the local 4–5-month post-analytical storage policy.

Owing to differences in residual sample availability, evaluable specimen numbers varied across markers. For the ACPA-dominant group and autoantibody-negative diagnostic samples (NCs; defined below), respectively: anti-CS IgG, n = 50/62; anti-CS IgM, n = 43/57; anti-HSP60 IgG, n = 52/67; anti-HSP60 IgM, n = 48/66; anti-HSP70 IgG, n = 49/67; anti-HSP70 IgM, n = 51/66.

### 2.4. Assay Ceiling-Substitution and Value Censoring

For all standard-curve-based ELISA assays used in the routine diagnostic pAAb panel (predominantly ORGENTEC by Sebia kits), optical density readings at or above the assay’s upper limit of quantification (ULOQ) are not extrapolated beyond the calibration curve. Per our laboratory’s standard operating procedure, agreed with the requesting clinicians, such results are automatically right-censored and substituted with the manufacturer-assigned nominal target value of the kit’s highest standard; this substituted value is what is transferred into the laboratory information system (LIMS) and used in all downstream analyses. The same policy was applied to the in-house nAAb ELISAs: results above the highest calibrator were right-censored at its nominal value, whereas results below the lowest reliably quantifiable calibrator were retained as measured.

### 2.5. Principles of Serological Group Allocation

#### 2.5.1. Marker Positivity and Disease Associations

Reactivity-dominant samples were identified from the pattern and relative magnitude of disease-associated autoantibody reactivity across the diagnostic panel (Supplementary Table S2, Supplementary Figure S1, Supplementary Table S3, Supplementary Figure S2), as detailed below; each sample was assigned to a single serogroup according to the highest fold-above-cut-off reactivity among the qualifying markers. Because the anti-SSA/SS-A assay’s ULOQ (100.1 U/mL) and the RF assays’ shared ULOQ (500.5 U/mL) are reached before the most strongly reactive samples in these groups would otherwise plateau, the anti-SSA/SSB-dominant group’s fold-above-cut-off values are uniformly capped at 4.00, and 11–17 samples per RF isotype are similarly capped at 25.02-fold (Supplementary Figures S1 and S2); these capped values reflect this assay ceiling rather than genuine biological uniformity and should not be read as a precise measure of true reactivity magnitude. For the aPL AAb-dominant group, the fold-above-cut-off value was defined as the higher of the cardiolipin IgG/cut-off and β2-glycoprotein-I IgG/cut-off ratios [40,41,42]. Autoantibody-negative diagnostic samples—hereafter abbreviated NC for brevity, and not intended to imply a healthy reference population—were negative for all measured pAAbs, including the markers used for serogroup definition; the NC label therefore reflects autoantibody status only, not confirmed clinical health. Anti-SSA-, ACPA-, anti-dsDNA-, and aPL AAb-dominant profiles were considered suggestive of Sjögren’s syndrome (SjS), rheumatoid arthritis (RA), systemic lupus erythematosus (SLE), and antiphospholipid syndrome (APS), respectively. A marker was considered positive when its measured value exceeded the assay cut-off (Supplementary Table S1). Anti-SSA/SSB dominance specifically was based on a two-tier reflex testing algorithm: samples were first screened using the composite ENA screen, and only ENA-screen-positive samples were subsequently tested on the antigen-specific confirmatory panel (anti-SSA/Ro, anti-SSB/La, anti-Sm, anti-RNP, anti-Scl-70, anti-Jo-1; ORGENTEC by Sebia, Mainz, Germany); group allocation used the antigen-specific anti-SSA/Ro and/or anti-SSB/La result, not the composite screen result, which served only as the first-tier trigger. ANA positivity was recorded but not used as a classification criterion, given its high background prevalence and low specificity in ACPA-positive rheumatoid arthritis [4].

#### 2.5.2. Large-Group Classification

Two levels of serological grouping were defined. In the large-group classification, five mutually exclusive groups were assigned: (i) autoantibody-negative diagnostic samples (NC); (ii) ACPA (anti-CCP) dominant; (iii) aPL AAb dominant; (iv) anti-dsDNA dominant; and (v) anti-SSA/SSB dominant. aPL positivity was defined by aCL/β2-GPI IgG reactivity exceeding the assay-specific cut-off; both IgG and IgM isotypes were measured in parallel for every sample, with IgG used as the diagnostically more relevant isotype for classification [1,2,3].

Reactivity-dominant group assignment followed a tiered rule reflecting the retrospective, serology-only nature of the cohort: dominance was determined from the pattern and magnitude of autoantibody reactivity alone, without reference to diagnosis. ACPA (anti-CCP) IgG positivity took absolute precedence. Among the remaining, non-ACPA-positive samples, where both aPL and anti-dsDNA reactivity reached dominance level, the sample was assigned to whichever showed the greater elevation, expressed as a multiple of its own cut-off—since, absent clinical information, relative serological magnitude was the only available basis for prioritizing between two positive disease-associated markers. Anti-dsDNA reactivity took priority over anti-SSA/SSB reactivity once anti-dsDNA itself reached dominance level; ordinary, lower-level anti-dsDNA positivity did not override anti-SSA/SSB dominance, reflecting the low specificity of low-titer anti-dsDNA reactivity, which can arise transiently—for example, following an antecedent infection—rather than reflecting an SLE-associated autoimmune process [2,5,6]. Anti-SSA/SSB dominance was therefore assigned when anti-SSA/SSB reactivity reached dominance level and no sample qualified as ACPA-, aPL-, or dominance-level anti-dsDNA-dominant. Co-occurring positivity on a lower-priority marker below its own dominance threshold is captured in the detailed-group subdivisions (e.g., “aPL AAb dominant with concurrent anti-dsDNA reactivity,” “dsDNA dominant with Sjögren-related positivity”) without altering the primary group assignment. This algorithm reproduced the assigned large-group classification for all 200 samples in the consolidated dataset without exception.

#### 2.5.3. Sample Distribution Across Groups

Of the 200 samples, 70 (35.0%) were negative for all four classification markers (autoantibody-negative diagnostic samples), 100 (50.0%) were positive for exactly one marker (unambiguous assignment), and 30 (15.0%) were positive for two or more markers and required adjudication by the dominance-priority algorithm described above; the most common co-positive combinations were anti-dsDNA with anti-SSA/SSB (n = 12) and anti-dsDNA with aPL (n = 8). No sample was excluded from serogroup allocation because classification was ambiguous; the algorithm assigned exactly one serogroup to every sample.

#### 2.5.4. Rationale for the Classification Approach

This classification algorithm reflects our laboratory’s established diagnostic practice for interpreting multi-marker autoantibody panels in routine clinical use, and was defined prior to any data analysis for this study, not derived post hoc from the observed results. Expressing each marker’s reactivity as a multiple of its own validated diagnostic cut-off is standard clinical laboratory practice for communicating result magnitude to referring clinicians across markers with different units and reference ranges (analogous to reporting a result as “3× the upper limit of normal”), and was applied here on that same basis rather than as a novel statistical construct.

#### 2.5.5. Detailed-Group Classification

In the detailed-group classification, the five “large groups” were further subdivided according to co-occurring autoantibody positivity, yielding nine subgroups: autoantibody-negative diagnostic samples (NC); ACPA dominant with or without RF-reactivity; ACPA dominant with additional SARD-related pAAbs (anti-SSA/SSB, other ENA-panel reactivity, ANuA, or anti-dsDNA); aPL AAb dominant without anti-dsDNA reactivity; aPL AAb dominant with concurrent anti-dsDNA reactivity; anti-dsDNA dominant with or without ANA and/or ANuA positivity; anti-dsDNA dominant with anti-SSA/SSB or other ENA-panel reactivity; anti-SSA/SSB dominant with additional RF-reactivity; and anti-SSA/SSB dominant without additional RF-reactivity.

#### 2.5.6. RF Isotype Positivity Within the ACPA-Dominant Group

Within the ACPA-dominant group (n = 53), RF seropositivity was assessed for the IgA, IgM, and IgG isotypes using the established cut-off of ≥20 U/mL (ORGENTEC by Sebia, Mainz, Germany). RF IgA positivity was detected in 53/53 samples (100%), while RF IgM and RF IgG positivity were each observed in 52/53 samples (98.1%) (Supplementary Figure S2, Supplementary Table S3).

### 2.6. Statistical Framework

#### 2.6.1. General Approach

Quantitative IgG/nAAb concentrations were log-transformed prior to regression modelling to reduce right-skew and stabilize variance. No nAAb value was zero or negative; log-transformation therefore required no offset. Wherever a quantitative outcome was regressed on a categorical or continuous predictor with age and sex as covariates, an ordinary least-squares model was fitted using heteroskedasticity-consistent (HC3) robust standard errors; fold-change estimates and 95% CIs were obtained by exponentiating the regression coefficients and their HC3-based confidence limits. Where distributional assumptions were assessed, the Shapiro–Wilk test was used; normality was not met in any tested variable (*p* < 0.001), so non-parametric methods (Spearman’s ρ, Mann–Whitney U) were used for the corresponding descriptive/exploratory analyses. Multiple comparisons within a pre-specified family of pairwise group contrasts were adjusted using the Holm step-down procedure; multiple comparisons within an exploratory family of correlations or group comparisons were adjusted using the Benjamini–Hochberg (BH) procedure, with Holm applied additionally as a more conservative sensitivity check where noted below. A two-sided adjusted *p* < 0.05 was considered statistically significant throughout.

#### 2.6.2. Age and Sex Distributions

**Figure 2:**
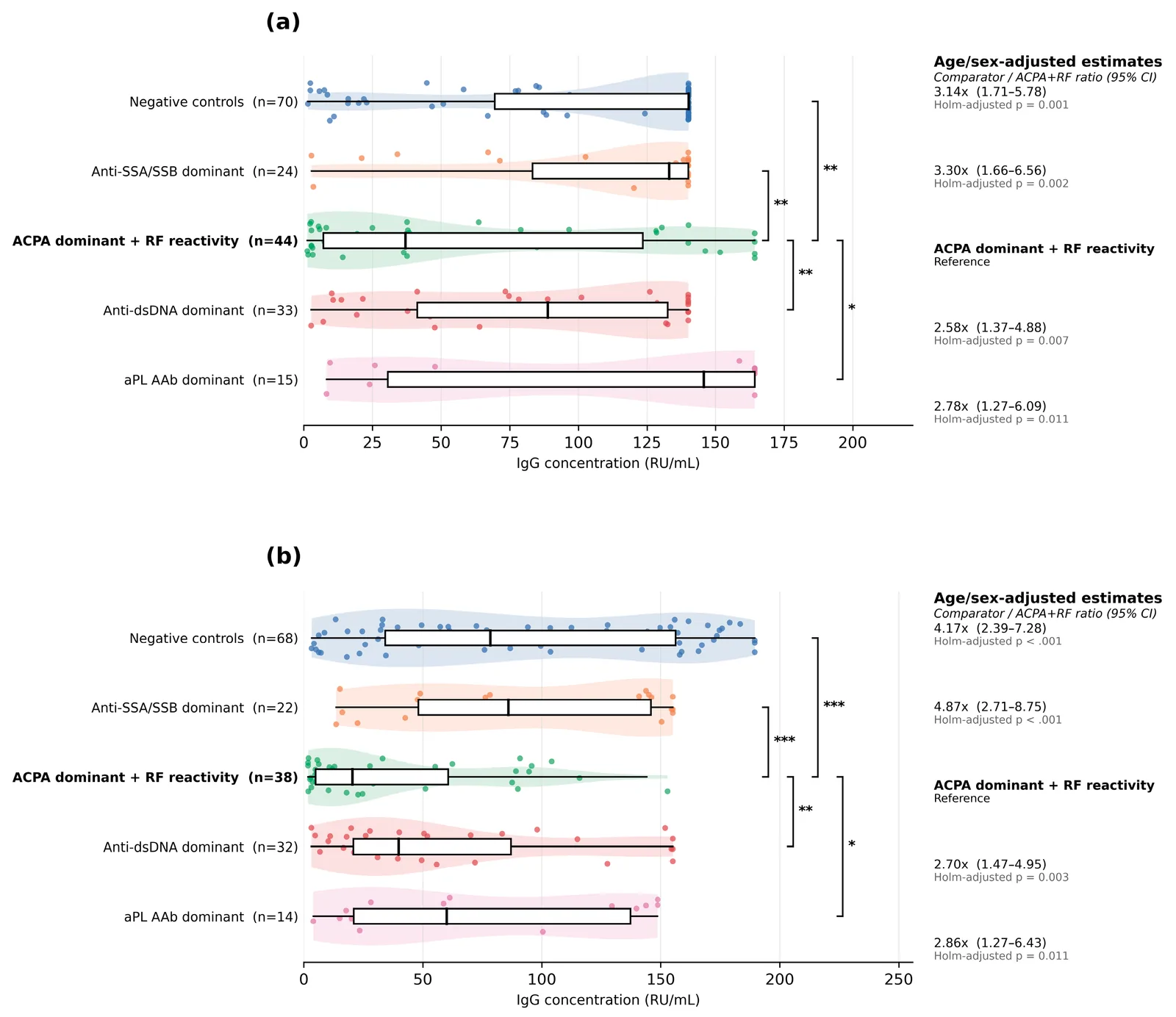
Quantitative SARS-CoV-2 S1-specific IgG by large serology groups. Raincloud plots showing the distribution of SARS-CoV-2 S1-specific IgG concentrations (RU/mL) across the five large, revised serology groups: NCs, anti-SSA/SSB dominant, ACPA dominant + RF reactivity (reference group, bold), anti-dsDNA dominant, and aPL AAb (antiphospholipid autoantibody) dominant. (**a**) QuantiVac (wild-type) SARS-CoV-2 S1-specific IgG (age/sex-adjusted overall serogroup effect: *p* = 0.005). (**b**) Omicron BA.1 SARS-CoV-2 S1-specific IgG (age/sex-adjusted overall serogroup effect: *p* < 0.001). Each row combines a kernel-density violin, an overlaid boxplot (median, IQR, whiskers to 1.5 × IQR), and individual jittered data points, colour-coded by group. Sample sizes (n) are shown per group; n differs slightly between the two assays owing to missing values. Age- and sex-adjusted fold-change estimates relative to the ACPA dominant + RF reactivity reference, their 95% confidence intervals, and Holm-adjusted *p*-values from log-linear regression models (HC3 robust standard errors) are shown to the right of each panel. Brackets connecting each comparator group to the reference indicate the significance of the pairwise contrast (* *p* < 0.05, ** *p* < 0.01, *** *p* < 0.001, ns = not significant, Holm-adjusted). All ACPA-dominant samples in this cohort were positive for at least one RF isotype (IgA, IgM, or IgG), so this group is designated ACPA dominant + RF reactivity rather than as a variable-RF-status group.

**Figure 3:**
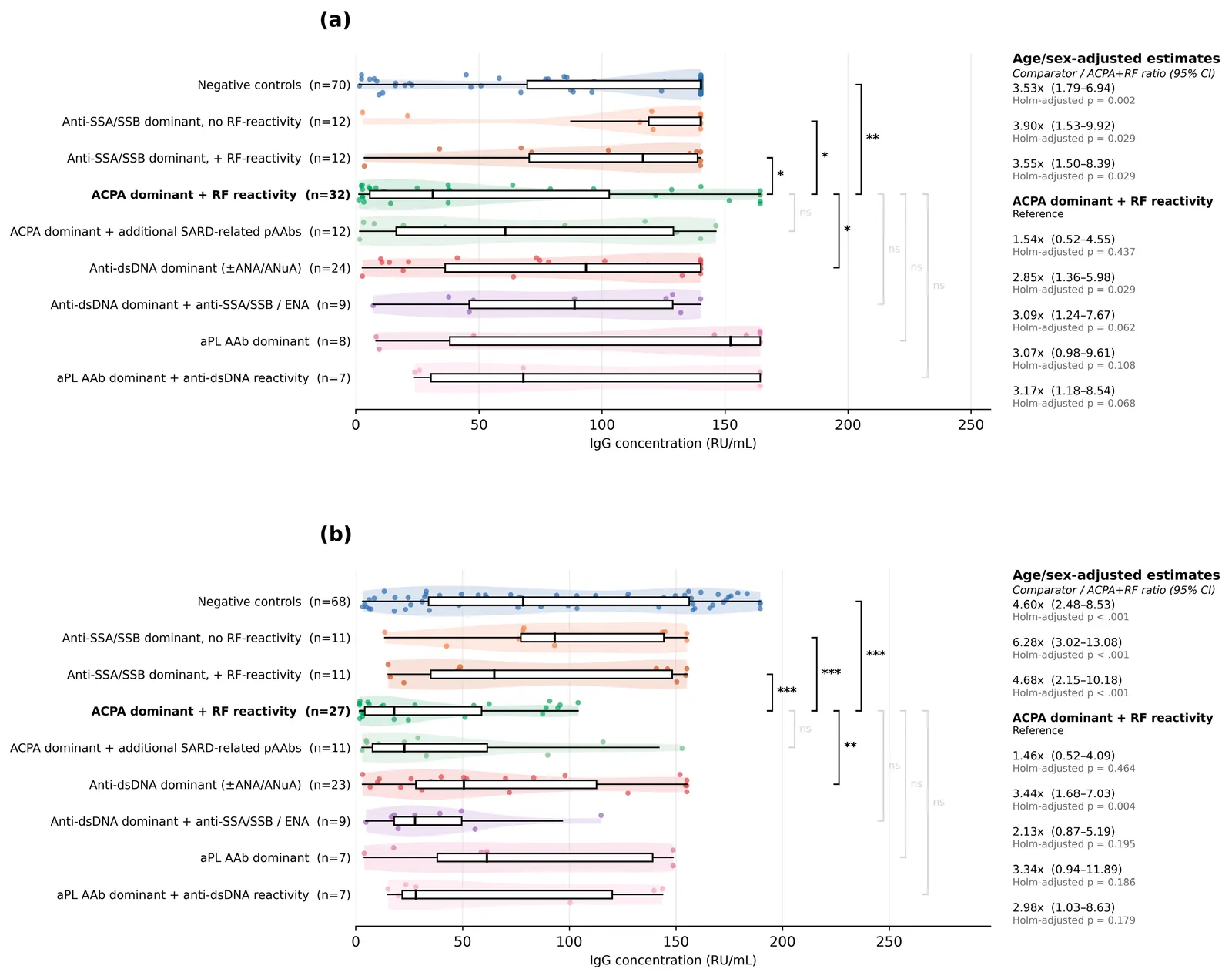
Quantitative SARS-CoV-2 S1-specific IgG by detailed serology groups (exploratory analysis). Raincloud plots as in Figure 2, but using the nine detailed revised serology subgroups: NCs, anti-SSA/SSB dominant without RF-reactivity, anti-SSA/SSB dominant with RF-reactivity, ACPA dominant + RF reactivity (reference group, bold), ACPA dominant with additional SARD-related pathogenic autoantibodies (pAAbs; anti-SSA/SSB, other ENA-panel reactivity, anti-nucleosome antibody [ANuA], or anti-dsDNA), anti-dsDNA dominant (with or without ANA and/or ANuA positivity), anti-dsDNA dominant with anti-SSA/SSB/ENA panel reactivity, aPL AAb dominant, and aPL AAb dominant with anti-dsDNA reactivity. (**a**) QuantiVac (wild-type) SARS-CoV-2 S1-specific IgG (age/sex-adjusted overall serogroup effect: *p* = 0.050). (**b**) Omicron BA.1 SARS-CoV-2 S1-specific IgG (age/sex-adjusted overall serogroup effect: *p* < 0.001). Plot elements, adjusted estimates, and significance brackets are as described for Figure 2, referenced against the ACPA dominant + RF reactivity group. Because several subgroups were small (n = 7–13), this figure should be interpreted as an exploratory, hypothesis-generating analysis rather than a confirmatory one.

Because the reactivity-dominant groups originated from routine diagnostic referrals rather than prospective age- or sex-matched recruitment, age and sex distributions were examined in every group-comparison analysis. Age differences were assessed using two-sided Mann–Whitney U tests against the ACPA-dominant reference, preceded by an omnibus Kruskal–Wallis test where more than two groups were compared; sex distributions were compared using two-sided Fisher’s exact tests. All pairwise age and sex comparisons were Holm-corrected within their respective family. Age and sex were additionally included as covariates in the regression sensitivity models described above. Detailed demographic characteristics for Figure 2 or Figure 3 and Figure 5 comparisons are provided in Supplementary Tables S4 and S5, respectively.

#### 2.6.3. Group Comparisons of SARS-CoV-2 IgG (Used for Figure 2 and Figure 3)

For each assay (QuantiVac, Omicron BA.1) and each grouping level (large, detailed), the regression outcome was log-transformed IgG concentration and the primary predictor was serology group (ACPA-dominant as reference). The overall effect of serology group was assessed using a Wald F-test. Pairwise contrasts against the ACPA-dominant reference were Holm-adjusted. Because several detailed subgroups were small (n = 7–13), the detailed-group analysis was treated as exploratory and hypothesis-generating, whereas the large-group analysis (n = 15–70 per group) served as the primary, confirmatory comparison. Sex information was complete for all samples; model sizes were n = 186 (QuantiVac) and n = 174 (Omicron BA.1).

#### 2.6.4. Correlation Analyses (Used for Figure 4)

Associations between (i) quantitative CovQV and CovOmi IgG and the pAAbs used in the RA diagnostic panel—ACPA IgG and the three RF isotypes (IgA, IgM, IgG), hereafter “RA-panel pAAbs”—and (ii) the six IgG/IgM nAAbs against CS, HSP60, and HSP70 and the same RA-panel pAAbs were assessed using two-sided Spearman’s rank correlation (ρ), with missing observations excluded pairwise and 95% CIs derived via the Fisher z-transformation. As a third analysis (iii), associations between the same six nAAbs and SARS-CoV-2 IgG (CovQV and CovOmi) directly were likewise assessed using two-sided Spearman’s rank correlation within the same population, with raw *p*-values BH-adjusted within each antiviral IgG assay’s family of six comparisons. ACPA is the more disease-specific marker for RA [40,41], whereas RF is a mechanistically nonspecific marker that can arise in numerous other contexts [42,43]; the two are grouped under a single label here only to describe which assays constitute the diagnostic panel, not to imply equivalent disease specificity. Both analyses were restricted to the ACPA-dominant + RF-reactivity group and NCs (n = 53 and 70, respectively, before pairwise exclusion of missing values)—the same population used for Figure 2 or Figure 3 group-comparison analyses, to keep results directly comparable. Because each analysis constitutes its own family of hypothesis tests (8 tests for analysis i; 24 tests for analysis ii), raw *p*-values were BH-adjusted separately within each family, with Holm applied as a sensitivity analysis. Each correlation matrix was visualised as a Spearman’s ρ heatmap annotated with pairwise sample size, 95% CI, raw *p*-value, and BH-adjusted *p*-value. As a further sensitivity analysis, each correlation was additionally evaluated using log-linear regression with serological group status, age, and sex as covariates, with the same multiplicity correction applied. Because ACPA IgG and the three RF isotypes were strongly intercorrelated (analysis i), partial Spearman correlations of each of the four RA-panel pAAbs with SARS-CoV-2 IgG were additionally computed, controlling for the other three pAAbs, as a sensitivity analysis to assess whether any single marker was associated with SARS-CoV-2 IgG independently of the others. Partial correlations were obtained by rank-transforming all variables, regressing the pAAb of interest and the outcome on the remaining three (rank-transformed) pAAbs, and correlating the residuals (Pearson’s r on residual ranks); significance was assessed via the corresponding t-distribution with n − 5 degrees of freedom (Supplementary Table S6). Because anti-HSP60 IgG and anti-HSP70 IgG were the most strongly intercorrelated nAAb pair (ρ = 0.48), the same partial-correlation approach was applied to this pair as a secondary, exploratory check of their associations with SARS-CoV-2 IgG (Supplementary Table S6).

#### 2.6.5. Group Comparisons of nAAb Reactivity (Used for Figure 5)

Differences in nAAb reactivity between the ACPA-dominant group and NCs were evaluated using two-sided Mann–Whitney U tests for each of the six nAAb reactivities. Effect sizes were expressed as rank-biserial correlations (rᵣᵦ) with bootstrap 95% CIs (5000 resamples), coded so that positive values indicate higher levels in the ACPA-dominant group. Because the six comparisons constitute a single family of tests, raw *p*-values were additionally BH-adjusted. Associations between age and each nAAb reactivity were examined using pooled Spearman correlation, BH-adjusted across the six comparisons, followed by an age- and sex-adjusted regression sensitivity analysis (as described above) yielding Holm-adjusted fold-differences relative to NCs for the pre-specified group contrast.

#### 2.6.6. Regression Diagnostics

Regression diagnostics were performed for all models described above. Variance inflation factors did not exceed 2.7 for any predictor in any model, indicating no material multicollinearity among serological group, age, and sex. Cook’s distance identified no observation with outsized influence on model estimates (maximum Cook’s D = 0.14, well below the conventional concern threshold of 1) in any model. Residuals departed from normality in most models (Shapiro–Wilk *p* < 0.05), consistent with the use of heteroskedasticity-consistent (HC3) standard errors throughout rather than reliance on the normality assumption.

#### 2.6.7. Sample-Size Considerations

Sample sizes were determined by the availability of specimens meeting the pAAb-based serogroup and residual-volume eligibility criteria described above, rather than by prospective power calculation; this is an inherent limitation of a retrospective, residual-specimen design. To contextualize the precision of the reported estimates, retrospective minimum detectable effects (80% power, two-sided α = 0.05) were calculated post hoc from the achieved group sizes and observed residual variance. For the primary large-group comparisons (Figure 2 or Figure 3), the study had 80% power to detect fold-differences of approximately 2.0-to-2.9-fold between the ACPA-dominant group and each comparator, below the 2.6-to-4.9-fold differences actually observed, indicating adequate power for the confirmatory analysis. For the smallest detailed subgroups (n = 7), the detectable fold-difference rose to approximately 4.3-fold, underscoring why the detailed-group analysis is treated as exploratory rather than confirmatory. For Figure 5 nAAb comparisons, the detectable fold-difference was approximately 1.6-fold for the smallest comparison (anti-CS IgM, n = 43/57); the one non-significant comparison in this panel (anti-CS IgG, adjusted ratio 1.26) falls below this threshold, consistent with insufficient power rather than a true absence of effect. These figures indicate the precision achieved rather than a prospective sample-size justification, and results—particularly for the detailed subgroups—should be interpreted with awareness of the risk of both false-negative and unstable effect estimates.

### 2.7. Visualization and Software

Raincloud plots (kernel-density violin, boxplot with median/IQR/1.5 × IQR whiskers, and jittered individual points) were used to visualise IgG and nAAb concentration distributions; correlation matrices were visualised as annotated heatmaps. All statistical analyses and figures were generated using OriginPro 8.5 (OriginLab Corporation, Northampton, MA, USA), Python (version 3.11.15); pandas (3.0.2), NumPy (2.4.4), SciPy (1.17.1), and Matplotlib (3.10.9). During the revision, selected figure-generation and statistical-analysis code was further refined with AI coding assistance (Visual Studio Code, version 1.141, Python 3.14); full disclosure of the tool and its use is provided in the Acknowledgments.

## 3. Results

### 3.1. Positivity Patterns of RA-Associated Serological Markers in the Regional Diagnostic Cohort

Before examining the ACPA-reactive substudy in detail, we first asked how common ACPA and the other pAAbs are in our routine diagnostic setting; this establishes the background against which the ACPA-reactive profile is compared in the sections that follow. To answer this, we examined autoantibody positivity patterns in the decade-long regional autoimmune-diagnostics dataset spanning 2014–2024 (Supplementary Table S1; Figure 1). Among the high-volume screening and supportive assays, the broad ANA screen [43,44]- showed the highest positivity rate, with 7245 positive individuals among 41,764 individuals tested (17.35%), followed by the diagnostically supportive, still less disease-specific, RF isotypes [9]: RF IgM (3155/24,515; 12.87%), RF IgG (3005/24,391; 12.32%), and RF IgA (2866/24,521; 11.69%). Among the more disease-specific autoantibody assays, anti-dsDNA IgG showed the highest positivity frequency (2447/27,118; 9.02%), followed by ACPA IgG (2041/26,437; 7.72%). However, low-level anti-dsDNA reactivity requires cautious interpretation, particularly near the assay cut-off, because borderline or transient positivity may occur outside established SLE [45,46]. This caveat is not specific to anti-dsDNA IgG: because positivity throughout this panel was assigned from any single measurement exceeding the assay-specific cut-off at any time during the decade-long observation period (see above), borderline or transient elevations could analogously affect other assays, including the ANA screen and RF isotypes. No sensitivity analysis required confirmatory positivity on a second, independent sample was performed for any assay; this is noted as a limitation applicable to the diagnostic-positivity analysis as a whole, not solely to anti-dsDNA IgG. Accordingly, when both diagnostic specificity and positivity frequency were considered, ACPA IgG showed the highest positivity frequency among the more disease-specific assays in this diagnostic cohort [47,48]. This is descriptive laboratory context and motivated the focus on ACPA-reactive serology in the subsequent analyses; it does not, by itself, establish that the ACPA-reactive residual samples selected for the serological substudy are representative of a clinically confirmed RA population, since diagnostic test positivity in a referred cohort is additionally shaped by test-ordering practices, referral patterns, and repeat testing. These positivity proportions should not be interpreted as general-population or disease prevalence.

### 3.2. Quantitative SARS-CoV-2 S1-Specific IgG Across Large Serology Groups (Primary Analysis)

Quantitative SARS-CoV-2-specific IgG distributions differed across the five large autoantibody-defined serology groups for both assays (Figure 2). The ACPA dominant + RF reactivity group showed markedly lower IgG concentrations than the negative-control reference and the other three comparator groups; notably, all samples in this group were positive for at least one RF isotype in addition to CCP, so no ACPA-dominant-without-RF subgroup was present in this cohort. For QuantiVac IgG, the median concentration was 37.0 RU/mL in the ACPA-dominant + RF reactivity group, compared with 140.2 RU/mL in the negative-control group, 133.1 RU/mL in the anti-SSA/SSB-dominant group, 88.8 RU/mL in the anti-dsDNA-dominant group, and 145.7 RU/mL in the aPL AAb-dominant group. For Omicron BA.1-specific IgG, the median concentration was 20.4 RU/mL in the ACPA-dominant + RF reactivity group compared with 78.4, 85.9, 39.8, and 60.0 RU/mL, respectively, in the four comparator groups.

#### Age- and Sex-Adjusted Model (Primary Analysis)

Because age distributions differed among the serological groups, quantitative IgG levels were subsequently evaluated in age- and sex-adjusted models. The overall serogroup effect was significant for both QuantiVac IgG (*p* = 0.005) and Omicron BA.1-specific IgG (*p* < 0.001). All comparator groups showed significantly higher adjusted IgG levels than the ACPA dominant + RF reactivity group for both assays. For QuantiVac IgG, adjusted levels relative to this reference were 3.14-fold higher in the negative-control group (95% CI 1.71–5.78; Holm-adjusted *p* = 0.001), 3.30-fold higher in the anti-SSA/SSB-dominant group (95% CI 1.66–6.56; adjusted *p* = 0.002), 2.58-fold higher in the anti-dsDNA-dominant group (95% CI 1.37–4.88; adjusted *p* = 0.007), and 2.78-fold higher in the aPL AAb-dominant group (95% CI 1.27–6.09; adjusted *p* = 0.011). For Omicron BA.1-specific IgG, adjusted levels were 4.17-fold higher in the negative-control group (95% CI 2.39–7.28; *p* < 0.001), 4.87-fold higher in the anti-SSA/SSB-dominant group (95% CI 2.71–8.75; *p* < 0.001), 2.70-fold higher in the anti-dsDNA-dominant group (95% CI 1.47–4.95; *p* = 0.003), and 2.86-fold higher in the aPL AAb-dominant group (95% CI 1.27–6.43; *p* = 0.011).

Thus, in this primary, better-powered comparison across the five large serology groups (n = 15–70 per group), an ACPA-dominant + RF reactivity serological profile was consistently and significantly associated with lower quantitative SARS-CoV-2 S1-specific IgG for both assays, and this association persisted after adjustment for age and sex.

### 3.3. Quantitative SARS-CoV-2 S1-Specific IgG Across Detailed Serology Subgroups (Exploratory Analysis)

To explore whether this association was uniform across ACPA-dominant + RF reactivity samples or modified by co-occurring autoantibody positivity, we further subdivided the large serology groups into nine detailed profiles, including a distinct subgroup of aPL AAb-dominant samples with concurrent anti-dsDNA reactivity. Because several of these subgroups were small (n = 7–13), this analysis is presented as exploratory and hypothesis-generating rather than confirmatory, and the findings should be interpreted with appropriate caution pending replication in a larger cohort.

Quantitative SARS-CoV-2-specific IgG distributions again differed across the nine detailed autoantibody-defined serological profiles for both assays (Figure 3). The ACPA dominant + RF reactivity group showed markedly lower IgG concentrations than the negative-control reference and most of the other comparator profiles. For QuantiVac IgG, the median concentration in this group was 31.3 RU/mL, compared with 140.2 RU/mL in the negative-control group, 140.1 RU/mL in the anti-SSA/SSB-dominant (no RF-reactivity) group, 116.6 RU/mL in the anti-SSA/SSB-dominant (+RF-reactivity) group, 93.5 RU/mL in the anti-dsDNA-dominant group, 88.8 RU/mL in the anti-dsDNA-dominant with anti-SSA/SSB/ENA panel reactivity group, 60.7 RU/mL in the ACPA dominant + additional SARD-related pAAbs group, 152.2 RU/mL in the aPL AAb-dominant group, and 68.0 RU/mL in the aPL AAb-dominant with anti-dsDNA reactivity group. A broadly similar pattern was observed for Omicron BA.1-specific IgG, for which the median concentration was 18.0 RU/mL in the ACPA dominant + RF reactivity group compared with 78.4, 93.1, 64.8, 50.5, 27.8, 22.8, 61.3, and 28.2 RU/mL, respectively, in the same eight comparator groups.

#### Age- and Sex-Adjusted Model (Exploratory Analysis)

In the age-adjusted model, the overall serogroup effect remained significant for QuantiVac IgG (*p* = 0.050) and was strongly significant for Omicron BA.1-specific IgG (*p* < 0.001). For QuantiVac IgG, adjusted levels relative to the ACPA dominant + RF reactivity reference were 3.53-fold higher in the negative-control group (95% CI 1.79–6.94; Holm-adjusted *p* = 0.002), 3.90-fold higher in the anti-SSA/SSB-dominant (no RF-reactivity) group (95% CI 1.53–9.92; adjusted *p* = 0.029), 3.55-fold higher in the anti-SSA/SSB-dominant (+RF-reactivity) group (95% CI 1.50–8.39; adjusted *p* = 0.029), and 2.85-fold higher in the anti-dsDNA-dominant group (95% CI 1.36–5.98; adjusted *p* = 0.029). The aPL AAb-dominant group (3.07-fold, 95% CI 0.98–9.61; *p* = 0.108), the aPL AAb-dominant with anti-dsDNA reactivity group (3.17-fold, 95% CI 1.18–8.54; *p* = 0.068), the anti-dsDNA-dominant with anti-SSA/SSB/ENA panel reactivity group (3.09-fold, 95% CI 1.24–7.67; *p* = 0.062), and the ACPA dominant + additional SARD-related pAAbs group (1.54-fold, 95% CI 0.52–4.55; *p* = 0.437) did not reach statistical significance after Holm adjustment.

For Omicron BA.1-specific IgG, adjusted levels were 4.60-fold higher in the negative-control group (95% CI 2.48–8.53; *p* < 0.001), 6.28-fold higher in the anti-SSA/SSB-dominant (no RF-reactivity) group (95% CI 3.02–13.08; *p* < 0.001), 4.68-fold higher in the anti-SSA/SSB-dominant (+RF-reactivity) group (95% CI 2.15–10.18; *p* < 0.001), and 3.44-fold higher in the anti-dsDNA-dominant group (95% CI 1.68–7.03; *p* = 0.004), all relative to the ACPA dominant + RF reactivity reference. The aPL AAb-dominant group (3.34-fold, 95% CI 0.94–11.89; *p* = 0.186), the aPL AAb-dominant with anti-dsDNA reactivity group (2.98-fold, 95% CI 1.03–8.63; *p* = 0.179), the anti-dsDNA-dominant with anti-SSA/SSB/ENA panel reactivity group (2.13-fold, 95% CI 0.87–5.19; *p* = 0.195), and the ACPA dominant + additional SARD-related pAAbs group (1.46-fold, 95% CI 0.52–4.09; *p* = 0.464) did not reach statistical significance.

Because several of the detailed subgroups were small—particularly the two aPL AAb-related subgroups (n = 7–8)—these non-significant comparisons should be regarded as inconclusive rather than as evidence of a true null effect; the wide confidence intervals observed (e.g., 95% CI 0.94–11.89 for the aPL AAb-dominant group, Omicron BA.1) reflect limited statistical power at this sample size rather than necessarily a smaller underlying effect.

Taken together, the exploratory detailed-group analysis raises the hypothesis that the reduced anti-SARS-CoV-2 IgG level seen in the primary ACPA dominant + RF reactivity comparison may not be uniform across all such samples, and that the aPL AAb-related groups follow a broadly similar pattern to the other comparator groups at the large-group level—while co-occurring autoantibody positivity (anti-SSA/SSB, other ENA-panel reactivity, ANuA, or anti-dsDNA) alongside ACPA/RF dominance, or concurrent anti-dsDNA reactivity within the aPL AAb-dominant group, may partially offset rather than compound these effects. Given the small subgroup sizes involved, this should be treated as a hypothesis for future, adequately powered studies rather than a confirmed finding.

Regression diagnostics indicated no material multicollinearity (VIF ≤ 2.64) or overly influential observations (Cook’s D ≤ 0.13) in either the large- or detailed-group models (Supplementary Table S7).

### 3.4. Associations Between SARS-CoV-2-Specific IgG Levels and RA-Panel pAAbs

**Figure 4:**
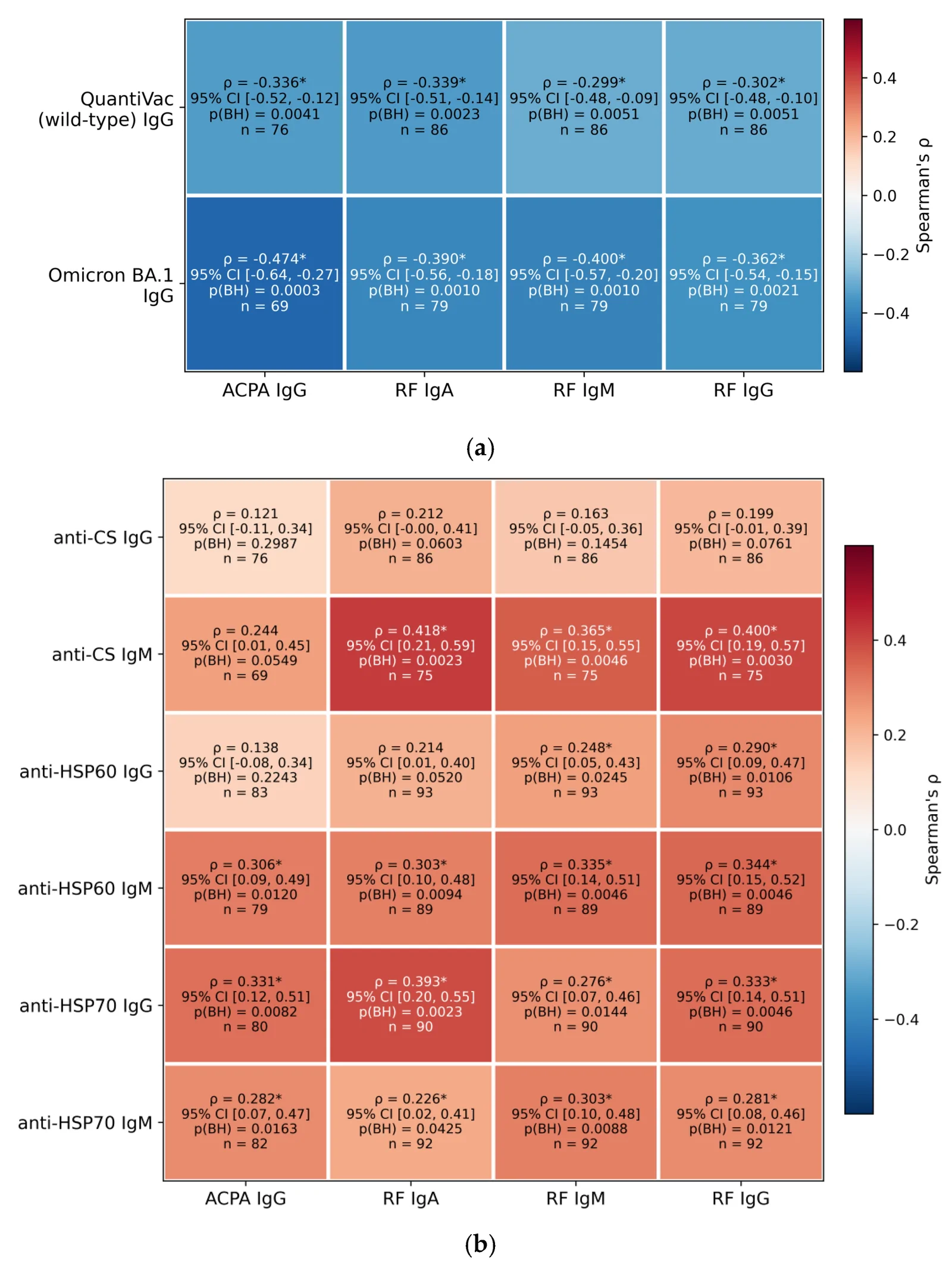
Association of SARS-CoV-2-specific IgG with pathological and natural autoantibodies in ACPA-reactive samples. Spearman correlation heatmaps restricted to ACPA IgG-reactive samples and autoantibody-negative reference samples (NCs; n = 53 and 70, respectively, before pairwise exclusion of missing values). (**a**) Associations between quantitative QuantiVac and Omicron BA.1-specific IgG levels and ACPA-associated pAAbs (ACPA IgG, RF IgA, RF IgM, RF IgG). (**b**) Associations between the six nAAb measures (anti-CS, anti-HSP60, anti-HSP70; IgG and IgM) and the same ACPA-associated pAAbs. In both panels, cell values show Spearman’s ρ, 95% confidence intervals (Fisher z-transformation), raw *p*-values, and Benjamini–Hochberg (BH)-adjusted *p*-values, together with pairwise sample sizes; asterisks denote adjusted *p* < 0.05 within each panel’s own family of tests (8 tests for panel (**a**); 24 tests for panel (**b**)). Cell shading follows the colour scale shown at right, with blue indicating negative and red indicating positive Spearman’s ρ; colour saturation increases with the magnitude of the correlation. All associations in panel (**a**) were inverse; all associations in panel (**b**) were positive. Two comparisons in panel (**b**) were nominally significant but did not survive BH correction, illustrating why multiplicity-adjusted values are reported as primary: anti-CS IgG vs. RF IgA (*p* = 0.050, p(BH) = 0.060) and anti-CS IgM vs. ACPA IgG (*p* = 0.043, p(BH) = 0.055). Age- and sex-adjusted sensitivity analyses (log-linear regression, HC3-robust standard errors) confirmed all eight associations in panel (**a**) and 20 of 24 associations in panel (**b**), with anti-CS IgG the consistent exception in panel (**b**), indicating these associations are not attributable to the age difference between ACPA-reactive samples and NCs.

As shown in Figure 4a, RA-panel pAAbs showed a consistent inverse relationship with QuantiVac IgG: ACPA IgG (ρ = −0.336, 95% CI −0.52 to −0.12, p(BH) = 0.0041, n = 76), RF IgA (ρ = −0.339, p(BH) = 0.0023, n = 86), RF IgM (ρ = −0.299, p(BH) = 0.0051, n = 86), and RF IgG (ρ = −0.302, p(BH) = 0.0051, n = 86). A concordant, numerically stronger pattern was observed for Omicron BA.1-specific IgG: ACPA IgG (ρ = −0.474, p(BH) = 0.0003, n = 69), RF IgA (ρ = −0.390, p(BH) = 0.0010, n = 79), RF IgM (ρ = −0.400, p(BH) = 0.0010, n = 79), and RF IgG (ρ = −0.362, p(BH) = 0.0021, n = 79). All eight associations remained significant after Holm correction (adjusted *p* ≤ 0.0095) and after adjustment for age and sex. The four RA-panel pAAbs were themselves strongly intercorrelated (ρ = 0.65–0.92); accordingly, as a sensitivity analysis, we computed the partial Spearman correlation of each marker with SARS-CoV-2 IgG after controlling for the other three (log-transformed). None of the four partial correlations remained statistically significant for either assay (QuantiVac: ACPA r = −0.070, *p* = 0.590; RF IgA r = −0.190, *p* = 0.120; RF IgM r = +0.110, *p* = 0.360; RF IgG r = −0.090, *p* = 0.450; Omicron BA.1: ACPA r = −0.200, *p* = 0.130; RF IgA r = −0.190, *p* = 0.150; RF IgM r = −0.040, *p* = 0.770; RF IgG r = +0.060, *p* = 0.660), indicating that the bivariate associations reported above cannot be attributed to any single marker independently of the others and most likely reflect a shared, collinear signal across the RA-panel pAAbs rather than four separable biological associations (Supplementary Table S6).

We additionally assessed direct associations between SARS-CoV-2 IgG and the six nAAbs. For Omicron BA.1 IgG, significant inverse correlations (BH-adjusted across the six comparisons) were observed with anti-CS IgG (ρ = −0.30, p(BH) = 0.006, n = 97), HSP60 IgG (ρ = −0.37, p(BH) < 0.001, n = 102), HSP70 IgG (ρ = −0.38, p(BH) < 0.001, n = 99), and HSP70 IgM (ρ = −0.24, p(BH) = 0.022, n = 102). For QuantiVac IgG, three of these four associations (HSP60 IgG, HSP70 IgG, and HSP70 IgM) were directionally concordant and reached only nominal significance (raw *p* = 0.014–0.037) but did not survive BH correction (p(BH) = 0.058–0.074); anti-CS IgG did not reach nominal significance for QuantiVac IgG (*p* = 0.066, p(BH) = 0.098). Because anti-HSP60 IgG and anti-HSP70 IgG were themselves moderately intercorrelated (ρ = 0.48), we additionally computed the partial correlation of each with SARS-CoV-2 IgG after controlling for the other (Supplementary Table S6). For Omicron BA.1 IgG, both associations remained nominally significant after mutual adjustment (HSP60 IgG: r = −0.28, *p* = 0.005; HSP70 IgG: r = −0.20, *p* = 0.046), indicating that, unlike the RA-panel pAAbs above, these two nAAbs are not fully accounting for one another’s association; for QuantiVac IgG, both associations were attenuated below significance after mutual adjustment (*p* = 0.06–0.28).

### 3.5. Associations Between nAAbs and RA-Panel pAAbs

As shown in Figure 4b, all associations between the six nAAb measures and the RA-panel pAAbs were positive. The strongest were anti-HSP70 IgG with RF IgA (ρ = 0.393, p(BH) = 0.0023, n = 90) and anti-CS IgM with RF IgA (ρ = 0.418, p(BH) = 0.0023) and RF IgG (ρ = 0.400, p(BH) = 0.0030, n = 75). Anti-HSP60 IgM and anti-HSP70 IgM were each significantly associated with all three RF isotypes and, for anti-HSP60 IgM, with ACPA IgG (ρ = 0.306, p(BH) = 0.0120, n = 79); anti-CS IgM’s association with ACPA IgG was weaker and did not survive BH correction (ρ = 0.244, p(BH) = 0.055, n = 69). Anti-HSP60 IgG reached significance for RF IgM and RF IgG only. Anti-CS IgG showed the weakest pattern: its comparison with RF IgA was nominally significant (*p* = 0.050) but did not survive BH correction (p(BH) = 0.060), and no other anti-CS IgG association reached significance. Two comparisons in this panel therefore failed to survive BH correction despite nominal *p*-values below 0.05: anti-CS IgG vs. RF IgA and anti-CS IgM vs. ACPA IgG. Age- and sex-adjusted sensitivity analyses confirmed 20 of the 24 associations, with anti-CS IgG the consistent exception.

Regression diagnostics indicated no material multicollinearity (VIF ≤ 1.49) or overly influential observations (Cook’s D ≤ 0.14) in the sensitivity models (Supplementary Table S7).

### 3.6. Enhanced nAAb Reactivity Across Most Markers in ACPA-Reactive Samples

**Figure 5:**
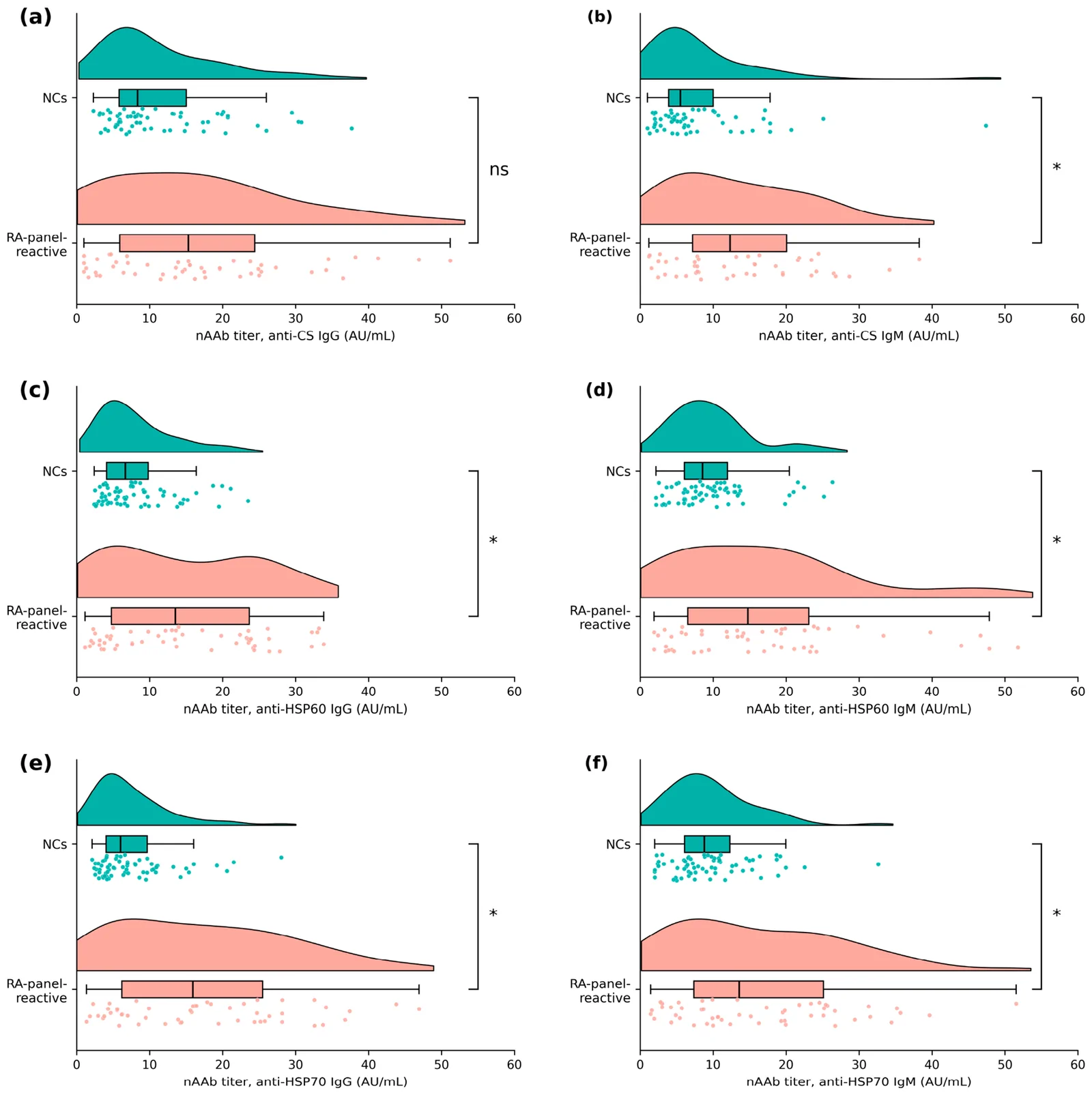
nAAb reactivity against citrate synthase (CS) and heat-shock proteins 60 and 70 (HSP60, HSP70) in RA-panel-reactive samples and autoantibody-negative reference samples (NCs). Raincloud plots showing serum IgG and IgM nAAb reactivities for (**a**) anti-CS IgG, (**b**) anti-CS IgM, (**c**) anti-HSP60 IgG, (**d**) anti-HSP60 IgM, (**e**) anti-HSP70 IgG, and (**f**) anti-HSP70 IgM (AU/mL). Group differences were assessed with two-sided Mann–Whitney U tests; effect sizes are reported as rank-biserial correlations (r_(_rb_)_) with bootstrap 95% confidence intervals (5000 resamples), coded so that positive values indicate higher levels in the RA-panel-reactive group. Raw *p*-values, effect sizes with 95% CIs, and pairwise sample sizes (RA-panel-reactive/NC) for all six comparisons are reported in the main text (Section 3.6.1 and Section 3.6.2); raw *p*-values were additionally adjusted across the six comparisons using the Benjamini–Hochberg (BH) procedure, and asterisks in the figure denote a BH-adjusted *p* < 0.05 (“ns” denotes a BH-adjusted *p* ≥ 0.05). BH-adjusted *p*-values were: anti-CS IgG, p(BH) = 0.067 (not significant); anti-CS IgM, p(BH) = 0.0012; anti-HSP60 IgG, p(BH) = 0.0025; anti-HSP60 IgM, p(BH) = 0.0028; anti-HSP70 IgG, p(BH) < 0.0001; anti-HSP70 IgM, p(BH) = 0.0028. Anti-CS IgG showed a higher median in the RA-panel-reactive group in the same direction as the other five nAAbs but did not reach significance; this is reported as not statistically significant rather than as a trend.

Given that higher SARS-CoV-2-specific IgG levels, particularly against the Omicron BA.1 variant, were inversely correlated with several nAAb reactivities (Figure 4), we next examined whether the selected nAAb profile differed between ACPA-reactive samples and NCs (Figure 5).

#### 3.6.1. IgG nAAbs

RA-panel-reactive samples exhibited consistently higher IgG nAAb levels than NCs. Effect sizes are reported as rank-biserial correlations (r_(_rb_)_), coded so that positive values indicate higher levels in the RA-panel-reactive group; raw *p*-values were additionally adjusted across all six nAAb comparisons using the BH procedure. For anti-CS IgG, RA-panel-reactive samples showed higher median values than NCs (15.4 vs. 8.35 AU); however, this difference did not reach statistical significance (*p* = 0.067, p(BH) = 0.067, r_(_rb_)_ = 0.20, 95% CI −0.03 to 0.42, n = 50/62). In contrast, anti-HSP60 IgG levels were significantly higher in RA-panel-reactive samples (median 13.64 vs. 6.67 AU; *p* = 0.0013, p(BH) = 0.0025, r_(_rb_)_ = 0.35, 95% CI 0.13–0.55, n = 52/67). Anti-HSP70 IgG showed the largest increase (median 16.18 vs. 6.01 AU; *p* < 0.001, p(BH) < 0.001, r_(_rb_)_ = 0.53, 95% CI 0.33–0.71, n = 49/67), the biggest IgG-associated difference among the investigated nAAbs.

#### 3.6.2. IgM nAAbs

The differences between RA-panel-reactive sera and NCs were more pronounced for the IgM isotype. Anti-CS IgM levels were significantly higher in RA-panel-reactive samples (median 12.75 vs. 5.50 AU; *p* < 0.001, p(BH) = 0.0012, r_(_rb_)_ = 0.42, 95% CI 0.19–0.62, n = 43/57). Anti-HSP60 IgM (median 15.78 vs. 8.55 AU; *p* = 0.0019, p(BH) = 0.0028, r_(_rb_)_ = 0.34, 95% CI 0.12–0.56, n = 48/66) and anti-HSP70 IgM (median 15.15 vs. 8.80 AU; *p* = 0.0024, p(BH) = 0.0028, r_(_rb_)_ = 0.33, 95% CI 0.11–0.53, n = 51/66) were also significantly elevated.

#### 3.6.3. Age and Sex Sensitivity Analysis of the nAAb Comparisons

Because RA-panel-reactive samples were derived from older individuals than NCs across all six nAAb comparisons, demographic effects were examined in additional sensitivity analyses (Supplementary Table S5). Sex distributions were comparable between groups in all panels. In pooled analyses, age was not significantly associated with five of the six nAAb reactivities after multiplicity correction; HSP70 IgG showed a positive age association (ρ = 0.316, 95% CI 0.14–0.47, adjusted *p* = 0.0033). Importantly, age- and sex-adjusted analyses preserved the overall between-group pattern. Relative to NCs, adjusted nAAb levels in RA-panel-reactive samples were 1.77-fold higher for CS-IgM (95% CI 1.16–2.70), 1.66-fold higher for HSP60-IgG (1.18–2.35), 1.67-fold higher for HSP60-IgM (1.24–2.25), 1.94-fold higher for HSP70-IgG (1.40–2.68), and 1.63-fold higher for HSP70-IgM (1.17–2.29); all remained significant after Holm correction. CS-IgG remained non-significant (adjusted ratio 1.26, 95% CI 0.89–1.79). Neither age nor sex was a significant covariate in any of the six adjusted models.

Regression diagnostics indicated no material multicollinearity (VIF ≤ 1.52) or overly influential observations (Cook’s D ≤ 0.10) in these models (Supplementary Table S7). The non-significant anti-CS IgG comparison (adjusted ratio 1.26) fell below this analysis’s retrospective minimum detectable effect (~1.6-fold; Supplementary Table S7), consistent with insufficient power rather than a demonstrated absence of effect.

## 4. Discussion

### 4.1. Beyond RA: Rheumatoid Factor as a Non-Specific Marker in SLE and Sjögren’s Syndrome

Rheumatoid factor (RF) is frequently assumed to be RA-specific, but it is a well-documented, non-specific finding across systemic autoimmune rheumatic diseases. In systemic lupus erythematosus (SLE) patients, RF positivity has been reported to be elevated in up to 27.7% of patients [49,50]. RF-positive SLE patients also appear to form a distinct serological cluster, with a higher prevalence of anti-Ro/SSA antibodies and, interestingly, relative protection from glomerulonephritis [51]. RF is even more prevalent in primary Sjögren’s syndrome (pSS): in one cohort, 66.7% of pSS patients were RF-positive compared with only 9.9% who were anti-CCP-positive [52]. A review of anti-CCP diagnostic performance likewise notes explicitly that high rates of false-positive RF occur in SLE and Sjögren’s syndrome, as well as chronic hepatitis [52].

Mechanistically, RF is not immunologically uniform: RF derived from Sjögren’s-associated disease preferentially binds distinct IgG-Fc epitopes compared with RA-associated RF, and RF reactivity in Sjögren’s patients is closely linked to the presence of anti-SSA/Ro antibodies, whereas RA-type RF reactivity coincides with anti-CCP—indicating that RF positivity accompanying ANA/dsDNA/SSA/SSB seropositivity may represent a biologically distinct, non-RA-associated antibody response rather than incidental co-positivity [53]. Consistent with this, RA–Sjögren’s overlap is a recognized clinical entity in which RF, anti-CCP, and anti-SSA are each independent risk factors for the overlap syndrome, with anti-SSA being the strongest predictor of RA patients later developing Sjögren’s [54].

Diagnostically, anti-CCP substantially outperforms RF in specificity for RA (RF specificity in the range of 70–85% vs. anti-CCP specificity around 95–97% in meta-analytic pooled data), while sensitivities are broadly comparable [55].

This asymmetry means an RF-positive/anti-CCP-negative serological pattern carries much less diagnostic weight for RA than either marker positive together [56,57], and—particularly when accompanied by ANA, anti-dsDNA, and/or anti-SSA/SSB positivity [51,53,54]—is more consistent with lupus- or Sjögren’s-associated RF, or a genuine RA–SLE/RA–Sjögren’s overlap, than with a distinct “CCP-seronegative RA” subgroup [58]. Definitive discrimination, however, requires clinical correlation (symmetric erosive synovitis, sicca features, renal/hematologic/dermatologic lupus manifestations) that serology alone cannot provide [54].

### 4.2. RA-Associated Serological Prominence in the Regional Diagnostic Cohort

Our routine diagnostic data supported the prominent position of RA-associated serology within the regional SARD spectrum. Although ANA showed the highest overall positivity, its broad screening nature limits disease specificity, while low-level anti-dsDNA IgG reactivity also requires cautious interpretation [45,46]. In contrast, ACPA IgG represented the most frequent highly disease-specific marker [47,48], accompanied by high RF positivity, consistent with the established serological prominence of RA [7,8]. These findings are consistent with published observations on the relative diagnostic specificity of ACPA versus RF and ANA, and provide descriptive laboratory context for the focus on ACPA-reactive serology in the subsequent analyses. They do not, however, establish that the ACPA-reactive residual samples selected for the serological substudy are representative of a clinically confirmed RA population; diagnostic test positivity in this referred cohort is additionally shaped by test-ordering practices, referral patterns, and repeat testing. As stated in the Methods, these positivity proportions reflect a referred diagnostic population and should not be interpreted as general-population or disease prevalence.

### 4.3. Distinct SARS-CoV-2-Specific Humoral Profile in ACPA-Reactive Samples

Direct quantitative comparison of SARS-CoV-2-specific IgG levels revealed a consistent separation of the ACPA-reactive serological profile from the other autoantibody-defined groups. ACPA-reactive samples showed lower IgG concentrations across both the QuantiVac and Omicron BA.1-specific assays, and this pattern remained after adjustment for age and sex. Relative to the ACPA-reactive group, adjusted IgG levels were 2.58–3.30-fold higher across the comparator groups for QuantiVac and 2.70–4.87-fold higher for Omicron BA.1-specific IgG, with all prespecified contrasts remaining significant after Holm correction. The concordance across two S1-specific assays therefore supports a robust cross-sectional association between ACPA-reactive serology and lower SARS-CoV-2-specific IgG magnitude, while not establishing the underlying clinical or biological cause of this difference. The exploratory detailed-group analysis (Figure 3) further suggests that this effect may not be uniform across all ACPA-reactive samples: the subgroup with additional SARD-related pAAb positivity showed a comparatively weaker, non-significant difference from the reference group (1.54-fold), in contrast to the significant differences observed for the pure ACPA-dominant and other detailed subgroups. Given the small size of this subgroup (n = 12), this pattern should be treated as a preliminary observation warranting confirmation rather than as evidence of a distinct biological subtype.

Reduced anti-Spike IgG and neutralizing-antibody responses after SARS-CoV-2 vaccination have repeatedly been reported in clinically defined RA cohorts, largely in association with immunomodulatory therapies, including methotrexate, B-cell depletion, JAK inhibitors and, in some studies, TNF inhibitors [6,12,13,59,60]. These observations provide relevant clinical context for the present findings but cannot be directly extrapolated to our cross-sectional serological dataset. Given the use of pseudonymised residual diagnostic samples and the absence of linked vaccination or infection histories, SARS-CoV-2-specific binding IgG was interpreted as an antiviral humoral readout without direct attribution to vaccine-induced immunity or clinical protection. This distinction is fundamental: the present study does not measure vaccine immunogenicity, since it captures SARS-CoV-2 S1-specific binding IgG at an unknown time after unknown, and potentially multiple, vaccination and/or infection exposures, in contrast to the cited studies, which typically involve confirmed diagnoses, known vaccine exposure, defined sampling intervals, and documented treatment status.

Accordingly, the added value of the present study lies not in another demonstration of reduced SARS-CoV-2-specific antibody levels in RA, but in identifying a coordinated serological context in which lower antiviral IgG is associated with broader RA-related autoreactivity spanning both ACPA/RF-defined pAAbs and selected nAAbs. The concordance of this pattern across two SARS-CoV-2 S1-specific IgG readouts, together with its persistence after adjustment for age and sex, indicates that this association is unlikely to be an artifact of a single assay or of age/sex confounding alone, although the cross-sectional design cannot establish whether it reflects a coordinated biological process or several independently co-occurring factors. Within this serology-centered framework, the principal features were the inverse associations between antiviral IgG and RA-associated autoreactivity and the enhanced nAAb profile, spanning most measured markers, of ACPA-reactive samples, as discussed below.

### 4.4. Lower SARS-CoV-2-Specific IgG Levels in ACPA-Reactive Samples After Adjustment for Age and Sex

The older age distribution of the ACPA-reactive group is biologically and diagnostically plausible within the real-world sampling framework of the study. RA-associated serology is predominantly encountered in adults and becomes increasingly relevant with age [4], whereas NCs were defined by the absence of measured pAAb reactivity rather than prospectively recruited as age-matched healthy controls. The NC group therefore represents a broader routine-diagnostic reference population that includes younger individuals investigated for diverse clinical indications. Similar demographic differences across the other serological profiles may likewise reflect underlying disease epidemiology and routine referral patterns. Importantly, no significant pairwise sex imbalance involving the ACPA-reactive group remained after multiplicity correction; neither age nor sex was independently associated with the antiviral IgG readouts in adjusted models, and the serogroup effect remained substantial across every comparator contrast. Thus, although residual demographic and unmeasured clinical confounding cannot be completely excluded, the observed age and sex differences do not readily account for the ACPA-associated serological separation. More broadly, selection bias should be considered explicitly rather than described only as a general feature of routine diagnostic sampling. All participants were drawn from a diagnostic laboratory population, and inclusion in the SARS-CoV-2 IgG/nAAb substudy additionally required sufficient residual serum volume. Both referral for autoimmune testing and the availability of residual serum may themselves be associated with underlying illness severity, clinical management decisions, or test-ordering patterns; if so, this selection process could introduce collider bias, whereby conditioning on a factor jointly influenced by disease-related characteristics and by the antibody measures under study induces or distorts associations that would not be present in an unselected population. This study cannot rule out such effects.

### 4.5. Inverse Relationship Between Antiviral IgG and RA-Associated Autoreactivity

Notably, both SARS-CoV-2 antibody readouts correlated inversely with ACPA IgG and all RF isotypes, while several nAAb reactivities showed concordant inverse relationships, particularly within the IgG compartment for Omicron BA.1-specific IgG, as reported above; the corresponding QuantiVac IgG associations were directionally consistent but weaker, reaching only nominal significance for three of the four markers and not surviving BH correction. Unlike the RA-panel pAAbs, for which none of the four markers retained an independent association after mutual adjustment, anti-HSP60 IgG and anti-HSP70 IgG—the most strongly intercorrelated nAAb pair (ρ = 0.48)—each retained a nominally significant partial association with Omicron BA.1 IgG after controlling for the other (Supplementary Table S6), suggesting that their associations are not simply redundant with one another, although this could not be confirmed for QuantiVac IgG.

Although direct evidence linking RF itself to lower SARS-CoV-2-specific IgG levels remains limited, RFs are well established to recognize the Fc region of IgG and to participate in IgG-containing immune complexes [8,10,11]. Because RF binds IgG directly, it could in principle alter the clearance, distribution, or assay-detectable concentration of IgG in circulation; the inverse relationships observed here may therefore be compatible with such differences in antiviral IgG handling or availability, providing a hypothesis-generating biological context for the observed serological pattern rather than a demonstrated mechanism. Analytical interference should also be considered, as RF can affect some SARS-CoV-2 serological assays, although such effects are assay-dependent and have not been consistently demonstrated with validated quantitative IgG assays [61]. This concern should be weighed equally alongside the immune-complex hypothesis above rather than treated as secondary: given that RF was positive in the great majority of ACPA-dominant samples, we cannot exclude that analytical interference, rather than biological IgG handling, contributes to the observed inverse relationship. Manufacturer validation data indicate no RF-related false-positive reactivity for the QuantiVac assay (0/40 RF-positive sera) but a low rate for the Omicron assay (2/37, 5.4%), and neither assay’s documentation specifies a dedicated RF-blocking reagent (see Methods); without independent interference-blocking or spike-and-recovery experiments in our own RF-positive samples, biological and analytical explanations cannot be distinguished. Furthermore, because ACPA and the three RF isotypes were strongly intercorrelated, a partial-correlation sensitivity analysis controlling each marker for the other three found no statistically significant independent association for any single marker (Results; Supplementary Table S6); this study therefore cannot determine whether ACPA, any individual RF isotype, or their shared variance underlies the observed inverse relationship with SARS-CoV-2 IgG. This does not weaken the central observation that ACPA/RF-dominant serology as a composite profile is associated with lower antiviral IgG, since that finding rests on the group-level comparisons (Figure 2 and Figure 3) rather than on distinguishing individual markers; it does mean, however, that the finding should be interpreted at the level of the overall autoreactive profile rather than attributed to ACPA or RF specifically.

### 4.6. Mechanistic Basis of the Enhanced nAAb Reactivity in ACPA-Reactive Samples

Furthermore, ACPA-reactive samples showed broadly enhanced nAAb reactivity across most markers, with significantly elevated anti-HSP60 and anti-HSP70 IgG and IgM levels, as well as anti-CS IgM, compared with NCs; the biggest between-group difference was observed for anti-HSP70 IgG, with anti-CS IgM showing the second-largest effect. Anti-CS IgG was the only marker that did not reach significance. This pattern is consistent with the expanding literature indicating that anti-HSP antibodies are frequently increased in RA and form part of a heterogeneous humoral response encompassing both potentially pathogenic and homeostatic immune functions [20,21,22,23,62]. Increased expression and extracellular release of HSPs during cellular stress, inflammation and tissue injury may enhance their availability as autoantigens, while the high degree of sequence conservation between microbial and human HSPs provides an additional mechanism for the generation or amplification of cross-reactive responses through molecular mimicry [20,22,62].

This group-level pattern was corroborated at the individual level: nAAb reactivities were also positively correlated with RA-panel pAAbs directly (Figure 4b), most consistently for anti-HSP70 IgG and anti-CS IgM with the RF isotypes. This is, to our knowledge, a novel observation and should be treated as hypothesis-generating: the cross-sectional design and the shared sample set with the group-level comparison (Figure 5) do not allow us to distinguish whether pathological and natural autoreactivity co-vary because they share upstream immunological drivers, or because both simply track overall autoimmune activity in this population.

### 4.7. ACPA-Associated nAAb Differences Are Not Readily Explained by Age or Sex

As discussed above, the ACPA-reactive group was older than the NC group, prompting additional evaluation of potential demographic confounding [4]. Importantly, age- and sex-adjusted sensitivity analyses preserved both the direction and magnitude of the ACPA-associated nAAb pattern, with approximately 1.6–1.9-fold higher adjusted levels of CS-IgM and HSP60/HSP70 IgG and IgM, while CS-IgG remained non-significant, closely reproducing the unadjusted nAAb pattern. Although HSP70 IgG showed an association with age in the pooled analysis, age was not independently associated with any of the nAAb levels after accounting for serological group and sex. These findings argue against age or sex alone explaining the ACPA-associated nAAb profile; however, unmeasured factors such as treatment, disease activity, or infection history were not available and cannot be excluded as contributors, so this pattern should be regarded as hypothesis-generating rather than as established evidence of a specific underlying autoreactive process.

## 5. Implications and Limitations

This study provides, to our knowledge, the first joint examination of pathological (ACPA/RF) and natural (CS/HSP-directed) autoreactivity in relation to antiviral IgG magnitude within the same referred diagnostic population, and several findings merit highlighting despite the exploratory nature of the analysis. First, the association between ACPA/RF-dominant serology and lower SARS-CoV-2-specific IgG was consistent across two independent antiviral assays and persisted after adjustment for age and sex, supporting it as a genuine, reproducible cross-sectional pattern rather than an assay-specific or purely demographic artifact. Second, and perhaps most notably, pathological and natural autoreactivity were not independent of one another: nAAb levels—particularly anti-HSP70 IgG and anti-CS IgM—correlated positively with the RA-panel pAAbs, in the same samples that showed reduced antiviral IgG. This concordance across three otherwise distinct serological readouts (antiviral IgG, pAAbs, and nAAbs) raises a specific, testable hypothesis: that pathological and natural autoreactivity may share upstream immunological drivers, or jointly track a common underlying process, rather than varying independently. We emphasize that this is a hypothesis generated by the present cross-sectional data, not a finding it can confirm; the associations described here, and the limitations discussed below, indicate that prospective, mechanistic studies are the appropriate next step.

### Limitations

The cross-sectional design precludes conclusions about causality. Clinical diagnoses, treatment, disease activity, and vaccination/infection history and timing were unavailable, so SARS-CoV-2 IgG reflects an antiviral readout rather than a vaccine-specific immunogenicity or protection endpoint, and vaccine- and infection-derived IgG cannot be distinguished. RA is typically managed with more intensive immunosuppression than the other profiles compared here, which is independently associated with attenuated antibody levels; because treatment data were unavailable, this cannot be excluded as an additional contributor to the ACPA group’s lower antiviral IgG.

The serological groups arose from routine diagnostic referrals rather than a population-based or prospectively recruited sample, and substudy eligibility required sufficient residual volume, making this a convenience rather than a random or consecutive sample. The study also lacks a healthy-donor control group: NCs are autoantibody-negative individuals from the same referred population, not prospectively recruited volunteers. The substudy spanned a wider age range (5–91 years) than the 18–80-year diagnostic-positivity cohort, reflecting its aim of capturing SARS-CoV-2 exposure across the whole population; age and sex were included as covariates throughout, and age was not independently associated with either antiviral IgG readout, but residual demographic and clinical confounding cannot be fully excluded.

The RA-panel pAAb composite combines ACPA IgG with the three RF isotypes, which differ substantially in specificity: ACPA is comparatively RA-specific, whereas RF is mechanistically nonspecific and can occur in infections, other autoimmune diseases, and healthy individuals with chronic antigenic stimulation. Because ACPA and RF were highly intercorrelated here (ρ = 0.65–0.92), associations attributed to “RA-panel pAAbs” may be driven disproportionately by RF and should not be read as RA-specific without further marker-level dissection. The proposed RF-mediated mechanisms (immune-complex formation, clearance, Fc-receptor interactions) remain hypothesis-generating, and RF/heterophile-antibody interference with the SARS-CoV-2 IgG assays could not be fully excluded.

Sample sizes reflected residual-specimen availability rather than prospective power calculation. The study was adequately powered for the primary large-group comparisons (detectable at 2.0- to 2.9-fold; observed differences 2.6- to 4.9-fold) but had limited power in the smaller detailed subgroups (n = 7–8) and the least-precise Figure 5 comparison; non-significant results in these settings should be considered inconclusive rather than evidence of no effect, and the detailed-group analysis (Figure 3) is presented as exploratory rather than confirmatory. Regression diagnostics did not identify material multicollinearity or overly influential observations in any model (Supplementary Table S7).

## 6. Conclusions

In this exploratory analysis of residual diagnostic samples, an ACPA-reactive serological profile was associated with lower SARS-CoV-2 S1-specific IgG levels and higher levels of selected CS- and HSP-directed autoantibodies. These cross-sectional associations persisted after adjustment for age and sex but cannot be separated from unmeasured effects of clinical diagnosis, immunomodulatory treatment, vaccination and infection history, sample timing, or potential analytical interference. Prospective studies involving clinically characterized participants and standardized post-vaccination sampling are required to determine the biological and clinical significance of these findings. Notably, the concordant, positive correlation observed here between natural and pathological autoreactivity—arising in the same samples that showed reduced antiviral IgG—offers a specific, testable hypothesis for such studies: that these two humoral compartments may share common upstream drivers rather than varying independently.

## Data Availability

The data supporting the findings of this study are not publicly available because they were derived from routine clinical diagnostic laboratory records and contain sensitive patient-related information subject to institutional data governance, ethical restrictions, and the General Data Protection Regulation (GDPR). The pseudonymised research dataset was generated from diagnostic data of 73,747 patients and remains under the custodianship of the Clinical Centre of the University of Pécs, Hungary. Data may be made available from the corresponding author upon reasonable request, subject to approval by the competent institutional bodies responsible for data protection and patients’ rights at the University of Pécs, and under conditions ensuring compliance with applicable legal, ethical, and institutional requirements. The study was approved by the relevant ethics committee (Ethical permission: 2-PTERKEB2026/1), and data processing was performed in collaboration with the Data Management Department of the Clinical Centre for research purposes only.

## Supplementary Materials

The following supporting information can be downloaded at https://www.mdpi.com/article/10.3390/vaccines14100888/s1, Table S1: Diagnostic pathological autoantibody (pAAb) positivity proportions (%) in the Southern Transdanubian regional autoimmune-diagnostics cohort, 2014–2024 (128,847 laboratory test episodes from 73,747 de-duplicated patients aged 18–80 years, comprising 405,948 individual assay results across the 14 assays below; see legend); Table S2: Fold-of-cut-off distribution of the group-defining pathological autoantibody (pAAb) marker(s) for the large and detailed serological groups (Figure 2 and Figure 3; Supplementary Figure S1); Table S3: Rheumatoid factor (RF) isotype positivity and fold-of-cut-off distribution in the ACPA-dominant + RF reactivity group (Supplementary Figure S2); Table S4: Demographic characteristics and demographic sensitivity analyses for the Figure 2 SARS-CoV-2-specific IgG cohorts; Table S5: Demographic characteristics and demographic sensitivity analyses for the Figure 5 nAAb comparisons; Table S6: Collinearity check: bivariate versus partial correlations of RA-panel pAAbs and selected nAAbs with SARS-CoV-2 IgG; Table S7: Regression diagnostics and retrospective sample-size/power characterization for all statistical models; Figure S1: Fold-of-cut-off distribution of the group-defining pathological autoantibody (pAAb) marker(s) for the large (a) and detailed (b) serological groups, with group sizes indicated (related to Table S2); Figure S2: Rheumatoid factor (RF) isotype (IgA, IgM, IgG) positivity and fold-of-cut-off distribution in the ACPA-dominant + RF reactivity group (n = 53; related to Table S3); Figure S3: Participant and specimen flow diagram, showing derivation of the regional autoimmune-diagnostics cohort (Panel A) and of the SARS-CoV-2 IgG/natural-autoantibody sub-study (Panel B).

## Abbreviations

**Abbreviation**	**Definition**
4-PL	Four-parameter logistic
aCL	Anticardiolipin antibody
ACPA	Anti-citrullinated protein antibody
ANA	Antinuclear antibody
ANCA	Antineutrophil cytoplasmic antibody
anti-dsDNA	Anti-double-stranded DNA antibody
aPL	Antiphospholipid antibody
APS	Antiphospholipid syndrome
a-PT	Anti-prothrombin antibody
AU	Arbitrary units
β2-GPI	β2-glycoprotein I
c-ANCA	Cytoplasmic antineutrophil cytoplasmic antibody
CCP	Cyclic citrullinated peptide
CI	Confidence interval
CovOmi	Quantitative Omicron BA.1-specific IgG variable
CovQV	Quantitative QuantiVac IgG variable
CS	Citrate synthase
ELISA	Enzyme-linked immunosorbent assay
ENA	Extractable nuclear antigen
Fc	Fragment crystallizable region
GDPR	General Data Protection Regulation
HC3	Heteroskedasticity-consistent type 3 robust standard errors
HEK293	Human embryonic kidney 293 cells
HSP	Heat-shock protein
HSP60	Heat-shock protein 60
HSP70	Heat-shock protein 70
IgA	Immunoglobulin A
IgG	Immunoglobulin G
IgM	Immunoglobulin M
JAK	Janus kinase
MPO	Myeloperoxidase
nAAb	Natural autoantibody
NC	Autoantibody-negative diagnostic sample
pAAb	Pathological autoantibody
p-ANCA	Perinuclear antineutrophil cytoplasmic antibody
PR3	Proteinase 3
PT	Prothrombin
RA	Rheumatoid arthritis
RF	Rheumatoid factor
RU	Reference units
SARD	Systemic autoimmune rheumatic disease
SARS-CoV-2	Severe acute respiratory syndrome coronavirus 2
SjS	Sjögren’s syndrome
SLE	Systemic lupus erythematosus
SSA	Sjögren’s syndrome-related antigen A (Ro/SSA)
TNF	Tumor necrosis factor
