# Supplementary material for: SARS-CoV-2-Specific IgG Levels Are Inversely Associated with ACPA-Reactive Pathological and Natural Autoantibody Profiles in Residual Diagnostic Samples
Source: Vaccines (Basel). 2026 Oct 10;14(10):888. doi: 10.3390/vaccines14100888 (PMC13709497; doi:10.3390/vaccines14100888)
Supplement: Supplementary file 1 [file vaccines-14-00888-s001.zip › Supplementary Table S1.pdf]

Supplementary Table S1. Diagnostic pathological autoantibody (pAAb) positivity proportions (%) in the Southern Transdanubian regional autoimmune-diagnostics cohort, 2014–2024 (128,847 laboratory test episodes from 73,747 de-duplicated patients aged 18–80 years, comprising 405,948 individual assay results across the 14 assays below; see legend).

Diagnostic pAAb Test	N tested	N Positive	Positivity (%)	CI low (%)	CI high (%)	Reference range, diagnostic threshold, manufacturer
ANA screen	41764	7245	17.347	16.987	17.714	2014–2017: REF < 10 U/mL HYCOR Biomedical LLC, USA 2017–2024: REF < 20 U/mL Werfen (Inova), USA
Anti-dsDNA IgG	27118	2447	9.024	8.688	9.37	REF <20 IU/mL Orgentec (Sebia) Mainz, Germany
Anti-nucleosome IgG	31590	2205	6.98	6.704	7.266	REF <20 U/mL Orgentec (Sebia) Mainz, Germany
ENA screen	35085	1824	5.199	4.971	5.436	2014–2017: REF < 10 U/mL HYCOR Biomedical LLC, USA, 2017–2024: REF < 1.0 U/mL Orgentec (Sebia) Mainz, Germany
aPL: aCL/β2-GPI IgM	30362	1412	4.651	4.419	4.893	aCL IgM REF < 7 U/mL;aβ2-GPI IgM REF < 8 U/mL Orgentec (Sebia) Mainz, Germany
aPL: aCL/β2-GPI IgG	30362	858	2.826	2.645	3.018	aCL IgG REF < 10 U/mL;aβ2-GPI IgG REF < 8 U/mL Orgentec (Sebia) Mainz, Germany
Anti-prothrombin IgG	22619	630	2.785	2.579	3.008	REF <10 U/mL Orgentec (Sebia) Mainz, Germany
Anti-prothrombin IgM	22619	367	1.623	1.466	1.796	REF <10 U/mL Orgentec (Sebia) Mainz, Germany

Anti-PR3 IgG (c-ANCA)	32283	342	1.059	0.953	1.177	Anti-MPO REF <10 RU/mL Orgentec (Sebia) Mainz, Germany
Anti-MPO IgG (p-ANCA)	32282	309	0.957	0.857	1.069	Anti-PR3 < 5 RU/mL Orgentec (Sebia) Mainz, Germany
RF IgM	24515	3155	12.87	12.456	13.296	REF <20 U/mL Orgentec (Sebia) Mainz, Germany
RF IgG	24391	3005	12.32	11.916	12.737	REF <20 U/mL Orgentec (Sebia) Mainz, Germany
RF IgA	24521	2866	11.688	11.293	12.095	REF <20 U/mL Orgentec (Sebia) Mainz, Germany
ACPA IgG	26437	2041	7.72	7.405	8.048	REF <25 U/mL SVAR LIFE SCIENCE INC, USA

*Patient records were de-duplicated and pseudonymized. Positivity was assigned according to the assay-specific diagnostic threshold and, where repeated measurements were available, based on the first measurement exceeding the respective cut-off. Positivity (%) was calculated as the proportion of positive individuals among all individuals tested for each assay. 95% confidence intervals (CIs) were calculated using the Wilson score method. The number of laboratory test episodes (128,847; i.e., individual blood draws/test orders) is smaller than the sum of the “N tested” column across all 14 assays (405,948) because a single test episode frequently included more than one assay (e.g., ANA, RF, and anti-dsDNA were often requested together from the same specimen); consequently, one episode can contribute to the “N tested” count of several different assays simultaneously, while the 73,747 de-duplicated patients reflect one count per unique patient (by national health insurance identifier, TAJ) irrespective of how many episodes or assays that patient underwent. Because this cohort comprises individuals referred for autoimmune diagnostic testing rather than a general-population sample, positivity proportions represent diagnostic test positivity rates within a referred clinical population and should not be interpreted as general-population or disease prevalence. N, number of individuals; pAAb, pathological autoantibody; ANA, antinuclear antibody; RF, rheumatoid factor; ACPA, anti-citrullinated protein antibody; dsDNA, double-stranded DNA; ENA, extractable nuclear antigen; aPL, antiphospholipid antibody; aCL, anticardiolipin; β2-GPI, β2-glycoprotein I; PR3, proteinase 3; MPO, myeloperoxidase; ANCA, antineutrophil cytoplasmic antibody; REF, reference range.
