# Supplementary material for: SARS-CoV-2-Specific IgG Levels Are Inversely Associated with ACPA-Reactive Pathological and Natural Autoantibody Profiles in Residual Diagnostic Samples
Source: Vaccines (Basel). 2026 Oct 10;14(10):888. doi: 10.3390/vaccines14100888 (PMC13709497; doi:10.3390/vaccines14100888)
Supplement: Supplementary file 1 [file vaccines-14-00888-s001.zip › Supplementary Table S2.pdf]

Supplementary Table S2. Fold-of-cut-off distribution of the group-defining pathological autoantibody (pAAb) marker(s) for the large and detailed serological groups (Figures 2 and 3; Supplementary Figure SF1).

A. Large serological groups

Group	n	Median fold	Mean fold	IQR (Q1–Q3)	Min	Max	Cut-off(s) applied (Supplementary Table S1)
Anti-SSA/SSB dominant	25	4	4	4.00–4.00	4	4	ENA screen: 2014–2017 REF <10 U/mL (HYCOR Biomedical LLC, USA); 2017–2024 REF <1.0 U/mL (Orgentec/Sebia, Mainz, Germany) [Supplementary Table S1]
ACPA dominant + RF reactivity	53	50.15	57.38	17.29–87.31	1.36	128.13	ACPA IgG REF <25 U/mL (SVAR LIFE SCIENCE INC, USA) [Supplementary Table S1]
Anti-dsDNA dominant	34	4.42	5.56	3.54–7.16	2.12	10.01	Anti-dsDNA IgG REF <20 IU/mL (Orgentec/Sebia, Mainz, Germany) [Supplementary Table S1]
aPL AAb dominant	18	4.98	6.2	3.60–8.51	2.54	12.51	aCL IgG <10 U/mL; aβ2-GPI IgG <8 U/mL (IgG used for fold calculation, per Methods); aCL IgM <7 U/mL; aβ2-GPI IgM <8 U/mL (reference only, not used in fold calculation) — Orgentec (Sebia), Mainz, Germany [Supplementary Table S1]

Note: Anti-SSA/SSB dominant fold values are uniformly 4.00 because all samples in this group reached the assay's upper calibration ceiling. Per laboratory protocol, optical density readings above the highest calibration standard are not extrapolated; results are automatically substituted with the manufacturer-assigned nominal target value of the top standard and transferred to the LIMS as this ceiling value. These fold values therefore represent a floor on, rather than a precise measure of, true anti-SSA/SSB reactivity magnitude (see Materials and Methods).

B. Detailed serological groups

Group	n	Median fold	Mean fold	IQR (Q1–Q3)	Min	Max	Cut-off(s) applied (Supplementary Table S1)
Anti-SSA/SSB dominant, no RF-reactivity	13	4	4	4.00–4.00	4	4	ENA screen: 2014–2017 REF <10 U/mL (HYCOR Biomedical LLC, USA); 2017–2024 REF <1.0 U/mL (Orgentec/Sebia, Mainz, Germany) [Supplementary Table S1]
Anti-SSA/SSB dominant, +RF-reactivity	12	4	4	4.00–4.00	4	4	ENA screen: 2014–2017 REF <10 U/mL (HYCOR Biomedical LLC, USA); 2017–2024 REF <1.0 U/mL (Orgentec/Sebia, Mainz, Germany) [Supplementary Table S1]
ACPA dominant + RF reactivity	41	59.45	62.25	18.82–95.04	4.35	128.13	ACPA IgG REF <25 U/mL (SVAR LIFE SCIENCE INC, USA) [Supplementary Table S1]
ACPA dominant + additional SARD-related pAAbs	12	40.01	40.73	13.48–55.17	1.36	128.13	ACPA IgG REF <25 U/mL (SVAR LIFE SCIENCE INC, USA) [Supplementary Table S1]
Anti-dsDNA dominant (±ANA/ANuA)	25	4.05	5.33	3.51–6.90	3.07	10.01	Anti-dsDNA IgG REF <20 IU/mL (Orgentec/Sebia, Mainz, Germany) [Supplementary Table S1]
Anti-dsDNA dominant + anti-SSA/SSB / ENA	9	5.92	6.18	4.01–8.19	2.12	10.01	Anti-dsDNA IgG REF <20 IU/mL (Orgentec/Sebia, Mainz, Germany) [Supplementary Table S1]
aPL AAb dominant	11	3.65	5.06	3.17–5.43	2.54	12.51	aCL IgG <10 U/mL; aβ2-GPI IgG <8 U/mL (IgG used for fold calculation, per Methods); aCL IgM <7 U/mL; aβ2-GPI IgM <8 U/mL (reference only, not used in fold calculation) — Orgentec (Sebia), Mainz, Germany

							[Supplementary Table S1]
aPL AAb dominant + anti-dsDNA reactivity	7	8.25	8	5.34–9.98	4.57	12.51	aCL IgG <10 U/mL; aβ2-GPI IgG <8 U/mL (IgG used for fold calculation, per Methods); aCL IgM <7 U/mL; aβ2-GPI IgM <8 U/mL (reference only, not used in fold calculation) — Orgentec (Sebia), Mainz, Germany [Supplementary Table S1]

Note: Anti-SSA/SSB dominant fold values are uniformly 4.00 because all samples in this group reached the assay's upper calibration ceiling. Per laboratory protocol, optical density readings above the highest calibration standard are not extrapolated; results are automatically substituted with the manufacturer-assigned nominal target value of the top standard and transferred to the LIMS as this ceiling value. These fold values therefore represent a floor on, rather than a precise measure of, true anti-SSA/SSB reactivity magnitude (see Materials and Methods).

Fold-of-cut-off was calculated as the patient value divided by the assay-specific diagnostic cut-off (Supplementary Table S1) for the marker defining each group's reactivity-dominance classification (Materials and Methods). n, number of samples in the group; IQR, interquartile range (25th–75th percentile); ACPA, anti-citrullinated protein antibody; RF, rheumatoid factor; dsDNA, double-stranded DNA; ENA, extractable nuclear antigen; aPL, antiphospholipid; AAb, autoantibody; aCL, anticardiolipin; β2-GPI, β2-glycoprotein I; ANA, antinuclear antibody; ANuA, anti-nucleosome antibody; SARD, systemic autoimmune rheumatic disease; REF, reference range.
