# Supplementary material for: SARS-CoV-2-Specific IgG Levels Are Inversely Associated with ACPA-Reactive Pathological and Natural Autoantibody Profiles in Residual Diagnostic Samples
Source: Vaccines (Basel). 2026 Oct 10;14(10):888. doi: 10.3390/vaccines14100888 (PMC13709497; doi:10.3390/vaccines14100888)
Supplement: Supplementary file 1 [file vaccines-14-00888-s001.zip › Supplementary Table S3.pdf]

Supplementary Table S3. Rheumatoid factor (RF) isotype positivity and fold-of-cut-off distribution in the ACPA-dominant + RF reactivity group (Supplementary Figure SF2).

RF isotype	n	n positive (>cut-off)	% positive	Median fold	Mean fold	IQR (Q1–Q3)	Min	Max	Cut-off(s) applied (Supplementary Table S1)
RF IgA	53	53	100	10.15	12.69	5.44–24.40	1.54	25.02	RF IgA/IgM/IgG REF <20 U/mL (Orgentec/Sebia, Mainz, Germany) [Supplementary Table S1]
RF IgM	53	52	98.1	18.57	17.16	10.50–25.02	0.2	25.02	RF IgA/IgM/IgG REF <20 U/mL (Orgentec/Sebia, Mainz, Germany) [Supplementary Table S1]
RF IgG	53	52	98.1	12.43	13.22	6.40–21.30	0.94	25.02	RF IgA/IgM/IgG REF <20 U/mL (Orgentec/Sebia, Mainz, Germany) [Supplementary Table S1]

Fold-of-cut-off was calculated as the patient value divided by the assay-specific diagnostic cut-off (Supplementary Table S1). n, number of samples in the ACPA-dominant + RF reactivity group (n = 53); n positive, number exceeding the isotype-specific cut-off; IQR, interquartile range (25th–75th percentile); ACPA, anti-citrullinated protein antibody; RF, rheumatoid factor; REF, reference range.
