# Supplementary material for: SARS-CoV-2-Specific IgG Levels Are Inversely Associated with ACPA-Reactive Pathological and Natural Autoantibody Profiles in Residual Diagnostic Samples
Source: Vaccines (Basel). 2026 Oct 10;14(10):888. doi: 10.3390/vaccines14100888 (PMC13709497; doi:10.3390/vaccines14100888)
Supplement: Supplementary file 1 [file vaccines-14-00888-s001.zip › Supplementary Table S4.pdf]

Supplementary Table S4. Demographic characteristics and demographic sensitivity analyses for the Figure 2 SARS-CoV-2-specific IgG cohorts

A. Age and sex distributions by serological group

Serological group	QuantiVac / CovQV IgG Quant cohort					Omicron BA.1 / CovOmi IgG Quant cohort				
**Serological group**	**n**	**Age, mean ± SD (years)**	**Age, median (IQR) (years)**	**Female, n/N (%)**	**Male, n/N (%)**	**n**	**Age, mean ± SD (years)**	**Age, median (IQR) (years)**	**Female, n/N (%)**	**Male, n/N (%)**
NC	70	39.8 ± 20.8	39.0 (20.0–52.8)	53/70 (75.7%)	17/70 (24.3%)	68	39.2 ± 20.7	39.0 (20.0–52.0)	52/68 (76.5%)	16/68 (23.5%)
anti-SSA/SSB-dominant	24	52.0 ± 14.4	51.5 (42.0–67.0)	22/24 (91.7%)	2/24 (8.3%)	22	50.5 ± 14.2	51.0 (42.0–60.8)	20/22 (90.9%)	2/22 (9.1%)
**ACPA-reactive**	**44**	**61.9 ± 14.7**	**64.5 (56.0–72.0)**	**35/44 (79.5%)**	**9/44 (20.5%)**	**38**	**63.6 ± 13.5**	**65.5 (57.0–72.0)**	**31/38 (81.6%)**	**7/38 (18.4%)**
anti-dsDNA-reactive	33	47.3 ± 17.0	46.0 (40.0–60.0)	32/33 (97.0%)	1/33 (3.0%)	32	46.7 ± 16.9	45.0 (38.2–57.8)	31/32 (96.9%)	1/32 (3.1%)
aPL-reactivity-dominant	15	51.2 ± 16.3	53.0 (44.0–57.0)	10/15 (66.7%)	5/15 (33.3%)	14	50.9 ± 16.8	53.0 (42.5–57.0)	9/14 (64.3%)	5/14 (35.7%)

B. Associations between age and quantitative SARS-CoV-2-specific IgG

Assay	Population	n	Spearman ρ	p	Interpretation
QuantiVac	All serogroups pooled	186	-0.048	0.511	Not significant
QuantiVac	ACPA-reactive	44	0.239	0.118	Not significant
QuantiVac	NC	70	0.151	0.212	Not significant
QuantiVac	anti-SSA/SSB-dominant	24	-0.343	0.101	Not significant
QuantiVac	anti-dsDNA-reactive	33	-0.019	0.914	Not significant
QuantiVac	aPL-reactivity-dominant	15	-0.069	0.806	Not significant
Omicron BA.1	All serogroups pooled	174	-0.109	0.153	Not significant
Omicron BA.1	ACPA-reactive	38	0.075	0.653	Not significant
Omicron BA.1	NC	68	0.082	0.504	Not significant
Omicron BA.1	anti-SSA/SSB-dominant	22	-0.074	0.745	Not significant
Omicron BA.1	anti-dsDNA-reactive	32	0.069	0.707	Not significant

Omicron BA.1	aPL-reactivity-dominant	14	0.024	0.934	Not significant
**C. Age- and sex-adjusted sensitivity analysis**					
**Assay**	**Model n**	**Overall serogroup effect p**	**Age p**	**Sex p**	**Interpretation**
QuantiVac	186	0.005	0.464	0.870	Serogroup effect retained; age and sex not significant
Omicron BA.1	174	<0.001	0.350	0.134	Serogroup effect retained; age and sex not significant
**D. Adjusted quantitative IgG differences relative to the ACPA-reactive group**					
**Assay**	**Comparator vs ACPA**	**Adjusted fold difference**	**95% CI**	**p**	**Holm-adjusted p**
QuantiVac	NC	3.14	1.71–5.78	<0.001	0.001
QuantiVac	anti-SSA/SSB-dominant	3.30	1.66–6.56	<0.001	0.002
QuantiVac	anti-dsDNA-reactive	2.58	1.37–4.88	0.004	0.007
QuantiVac	aPL-reactivity-dominant	2.78	1.27–6.09	0.011	0.011
Omicron BA.1	NC	4.17	2.39–7.28	<0.001	<0.001
Omicron BA.1	anti-SSA/SSB-dominant	4.87	2.71–8.75	<0.001	<0.001
Omicron BA.1	anti-dsDNA-reactive	2.70	1.47–4.95	0.002	0.003
Omicron BA.1	aPL-reactivity-dominant	2.86	1.27–6.43	0.011	0.011

Table legend. (A) Only samples with an available quantitative result for the respective SARS-CoV-2 IgG assay were included. Age is presented as mean ± SD and median (IQR); sex as n/N (%). Age distributions differed across serological groups in both cohorts (QuantiVac: Kruskal–Wallis H = 36.12, $p < 0.001$; Omicron BA.1: H = 39.30, $p < 0.001$), with ACPA-reactive samples older than each comparator after Holm correction (all adjusted $p < 0.05$). Sex distributions differed overall, but no significant pairwise sex difference involving the ACPA-reactive group remained after multiplicity adjustment. (B) Age–IgG relationships were assessed using two-sided Spearman rank correlation in the pooled cohorts and within individual serological groups. (C–D) To assess potential demographic confounding, quantitative IgG values were analysed in age- and sex-adjusted sensitivity models, with the ACPA-reactive group as the reference. Adjusted fold differences represent the relative quantitative IgG level in each comparator group versus the ACPA-reactive group after adjustment for age and sex; Holm correction was applied across the four comparator contrasts within each assay. Sex information was complete for all samples in both cohorts; model n = 186 for QuantiVac and n = 174 for Omicron BA.1. The serological groups originated from routine autoimmune-diagnostic referrals and were not prospectively age- or sex-matched; NCs were defined by negativity for all measured pathological autoantibodies rather than recruited as age-matched healthy

controls. Despite the older age distribution of the ACPA-reactive group, age was not significantly associated with either SARS-CoV-2-specific IgG readout, and the ACPA-associated serological separation persisted after demographic adjustment. NC, negative controls; ACPA, anti-citrullinated protein antibodies; aPL, antiphospholipid antibodies; SD, standard deviation; IQR, interquartile range; CI, confidence interval.
