# Supplementary material for: SARS-CoV-2-Specific IgG Levels Are Inversely Associated with ACPA-Reactive Pathological and Natural Autoantibody Profiles in Residual Diagnostic Samples
Source: Vaccines (Basel). 2026 Oct 10;14(10):888. doi: 10.3390/vaccines14100888 (PMC13709497; doi:10.3390/vaccines14100888)
Supplement: Supplementary file 1 [file vaccines-14-00888-s001.zip › Supplementary Table S5.pdf]

Supplementary Table S5. Demographic characteristics and demographic sensitivity analyses for the Figure 5 nAAb comparisons

A. Panel-specific age and sex distributions

Panel	nAAb	NC n	NC age, mean ± SD	NC age, median (IQR)	NC female, n/N (%)	ACPA n	ACPA age, mean ± SD	ACPA age, median (IQR)	ACPA female, n/N (%)	Age p	Age Holm-adjusted p	Sex p	Sex Holm-adjusted p
a	CS-IgG	62	38.5 ± 19.9	39.0 (20.0–48.5)	47/62 (75.8%)	50	61.8 ± 14.2	64.5 (56.0–71.0)	37/50 (74.0%)	<0.001	<0.001	0.830	1.000
b	CS-IgM	57	40.2 ± 19.5	39.0 (22.0–52.0)	43/57 (75.4%)	43	64.2 ± 11.6	66.0 (57.0–71.5)	31/43 (72.1%)	<0.001	<0.001	0.819	1.000
c	HSP60-IgG	67	39.5 ± 20.1	39.0 (20.0–52.5)	51/67 (76.1%)	52	62.0 ± 14.1	65.0 (56.0–71.2)	38/52 (73.1%)	<0.001	<0.001	0.832	1.000
d	HSP60-IgM	66	40.2 ± 20.1	39.0 (20.0–52.8)	51/66 (77.3%)	48	61.1 ± 14.4	64.5 (55.8–69.5)	36/48 (75.0%)	<0.001	<0.001	0.826	1.000
e	HSP70-IgG	67	39.5 ± 20.1	39.0 (20.0–52.5)	51/67 (76.1%)	49	61.1 ± 14.2	64.0 (56.0–69.0)	37/49 (75.5%)	<0.001	<0.001	1.000	1.000
f	HSP70-IgM	66	40.2 ± 20.1	39.0 (20.0–52.8)	51/66 (77.3%)	51	62.0 ± 14.2	65.0 (56.0–71.5)	37/51 (72.5%)	<0.001	<0.001	0.667	1.000

B. Associations between age and nAAb reactivity

Panel	nAAb	n	Spearman ρ	p	BH-adjusted p
a	CS-IgG	112	0.032	0.739	0.744
b	CS-IgM	100	0.198	0.048	0.143
c	HSP60-IgG	119	0.110	0.234	0.468
d	HSP60-IgM	114	0.031	0.744	0.744
e	HSP70-IgG	116	0.316	<0.001	0.003
f	HSP70-IgM	117	0.084	0.369	0.554

C. Age- and sex-adjusted model summary

Panel	nAAb	Model n	Serogroup effect p	Serogroup Holm-adjusted p	Age p	Sex p	Interpretation
a	CS-IgG	112	0.189	0.189	0.529	0.748	Adjusted group difference not significant

b	CS-IgM	100	0.008	0.017	0.916	0.631	ACPA-associated increase retained
c	HSP60-IgG	119	0.004	0.017	0.560	0.726	ACPA-associated increase retained
d	HSP60-IgM	114	<0.001	0.005	0.129	0.438	ACPA-associated increase retained
e	HSP70-IgG	116	<0.001	<0.001	0.305	0.822	ACPA-associated increase retained
f	HSP70-IgM	117	0.005	0.017	0.323	0.266	ACPA-associated increase retained

D. Adjusted nAAb differences between ACPA-reactive samples and NCs

Panel	nAAb	Adjusted ACPA/NC ratio	95% CI	p	Holm-adjusted p
a	CS-IgG	1.26	0.89–1.79	0.189	0.189
b	CS-IgM	1.77	1.16–2.70	0.008	0.017
c	HSP60-IgG	1.66	1.18–2.35	0.004	0.017
d	HSP60-IgM	1.67	1.24–2.25	<0.001	0.005
e	HSP70-IgG	1.94	1.40–2.68	<0.001	<0.001
f	HSP70-IgM	1.63	1.17–2.29	0.005	0.017

Table legend. (A) Only samples with a valid numeric result for the respective nAAb measurement were included in each panel-specific analysis. Age is presented as mean ± SD and median (IQR), and sex as n/N (%). Age differences were assessed using two-sided Mann–Whitney U tests and sex distributions using two-sided Fisher's exact tests; Holm correction was applied across the six Figure 5 panels. ACPA-reactive samples were older than NCs in all six comparisons, whereas sex distributions were comparable. (B) Pooled associations between age and nAAb reactivity were evaluated using two-sided Spearman rank correlation with Benjamini–Hochberg (BH) correction, consistent with the multiplicity procedure used for this comparison family in the main text. After correction, age was not significantly associated with five of the six nAAb reactivities; HSP70-IgG showed a positive age association. (C–D) To assess potential demographic confounding, log-transformed nAAb values were analysed in age- and sex-adjusted sensitivity models with serological group (ACPA-reactive versus NC), age, and sex included simultaneously. Adjusted ACPA/NC ratios are exponentiated serogroup coefficients and represent the relative nAAb level in ACPA-reactive samples compared with NCs after adjustment for age and sex; values >1 indicate higher adjusted levels in ACPA-reactive samples. Holm correction was applied across the six adjusted serogroup effects. The significant increases in CS-IgM and HSP60/HSP70 IgG and IgM were retained after demographic adjustment, whereas CS-IgG remained non-significant. Age and sex were not significant covariates in any of the six adjusted models. The groups arose from routine autoimmune-diagnostic sampling rather than prospective age- or sex-matched recruitment. The older age distribution of the ACPA-reactive group is compatible with the adult age distribution of RA-associated serology, while NCs were defined by absence of measured pathological autoantibody reactivity and therefore represent a broader routine-diagnostic reference population. Thus, although residual

confounding cannot be completely excluded, demographic differences alone do not readily account for the observed ACPA-associated nAAb pattern. NC, autoantibody-negative diagnostic samples; ACPA, anti-citrullinated protein antibodies; CS, citrate synthase; HSP, heat-shock protein; nAAb, natural autoantibody; SD, standard deviation; IQR, interquartile range; CI, confidence interval.
