# Supplementary material for: SARS-CoV-2-Specific IgG Levels Are Inversely Associated with ACPA-Reactive Pathological and Natural Autoantibody Profiles in Residual Diagnostic Samples
Source: Vaccines (Basel). 2026 Oct 10;14(10):888. doi: 10.3390/vaccines14100888 (PMC13709497; doi:10.3390/vaccines14100888)
Supplement: Supplementary file 1 [file vaccines-14-00888-s001.zip › Supplementary Table S6.pdf]

Supplementary Table S6. Collinearity check: bivariate versus partial correlations of RA-panel pAAbs and selected nAAbs with SARS-CoV-2 IgG.

A. RA-panel pAAbs (ACPA IgG, RF IgA, RF IgM, RF IgG) — primary collinearity check

**Antiviral IgG assay**	**Marker**	**Bivariate ϱ / r**	**Bivariate p**	**Partial r (controlling for other 3 pAAbs)**	**Partial p**
QuantiVac	ACPA IgG	-0.336	<0.001	-0.07	0.59
QuantiVac	RF IgA	-0.339	<0.001	-0.19	0.12
QuantiVac	RF IgM	-0.299	0.005	+0.11	0.36
QuantiVac	RF IgG	-0.302	0.005	-0.09	0.45
Omicron BA.1	ACPA IgG	-0.474	<0.001	-0.20	0.13
Omicron BA.1	RF IgA	-0.390	<0.001	-0.19	0.15
Omicron BA.1	RF IgM	-0.400	<0.001	-0.04	0.77
Omicron BA.1	RF IgG	-0.362	<0.001	+0.06	0.66

B. Selected nAAbs (HSP60 IgG, HSP70 IgG) — secondary check, given their moderate mutual correlation (ϱ = 0.48)

**Antiviral IgG assay**	**Marker**	**Bivariate ϱ**	**Bivariate p**	**Partial r (controlling for the other marker)**	**Partial p**
QuantiVac	HSP60 IgG	-0.234	0.014	-0.18	0.062
QuantiVac	HSP70 IgG	-0.226	0.019	-0.11	0.279
Omicron BA.1	HSP60 IgG	-0.365	<0.001	-0.28	0.005
Omicron BA.1	HSP70 IgG	-0.380	<0.001	-0.20	0.046

Table legend. (A) The four RA-panel pAAbs (ACPA IgG and the three RF isotypes) were strongly intercorrelated (ϱ = 0.65–0.92) within the ACPA-dominant + NC population (n = 123 before pairwise exclusion). Bivariate Spearman correlations of each marker with SARS-CoV-2 IgG (as reported in Figure 4a) are shown alongside the partial correlation of each marker with SARS-CoV-2 IgG after controlling for the other three pAAbs (rank-based residualization; see Materials and Methods). None of the four partial correlations reached statistical significance for either assay, indicating that the bivariate associations cannot be attributed to any single marker independently of the others. (B) Because anti-HSP60 IgG and anti-HSP70 IgG were the two most strongly intercorrelated nAAbs (ϱ = 0.48, versus ϱ = 0.65–0.92 for the pAAbs in panel A), the same partial-correlation approach was applied to this pair alone as a secondary, exploratory check. Unlike the pAAbs in panel A, both nAAbs retained part of their association with Omicron BA.1 IgG after mutual adjustment (nominally significant for both markers), indicating that — in contrast to ACPA/RF — the nAAb collinearity does not fully account for these associations, although some shared variance is present and the QuantiVac associations did not survive mutual adjustment. All other nAAb pairs were less strongly intercorrelated (ϱ = 0.08–0.44) and were not re-examined. ACPA, anti-citrullinated protein antibody; RF, rheumatoid factor; nAAb, natural autoantibody; NC, autoantibody-negative diagnostic sample.
