# Supplementary material for: SARS-CoV-2-Specific IgG Levels Are Inversely Associated with ACPA-Reactive Pathological and Natural Autoantibody Profiles in Residual Diagnostic Samples
Source: Vaccines (Basel). 2026 Oct 10;14(10):888. doi: 10.3390/vaccines14100888 (PMC13709497; doi:10.3390/vaccines14100888)
Supplement: Supplementary file 1 [file vaccines-14-00888-s001.zip › Supplementary Table S7.pdf]

Supplementary Table S7. Regression diagnostics and retrospective sample-size/power characterization for all statistical models.

A. Regression diagnostics by model

Model	n	Max VIF	Max Cook's D	Shapiro–Wilk p (residuals)
Fig2 (large-group), QuantiVac	186	2.00	0.065	<0.0001
Fig2 (large-group), Omicron BA.1	174	2.19	0.090	<0.0001
Fig3 (detailed-group), QuantiVac	186	2.36	0.073	<0.0001
Fig3 (detailed-group), Omicron BA.1	174	2.64	0.132	<0.0001
Fig4a, QuantiVac vs ACPA	76	1.13	0.141	0.0009
Fig4a, QuantiVac vs RF IgA	86	1.27	0.136	<0.0001
Fig4a, QuantiVac vs RF IgM	86	1.26	0.134	<0.0001
Fig4a, QuantiVac vs RF IgG	86	1.18	0.133	<0.0001
Fig4a, Omicron BA.1 vs ACPA	69	1.21	0.116	0.0035
Fig4a, Omicron BA.1 vs RF IgA	79	1.35	0.101	0.0036
Fig4a, Omicron BA.1 vs RF IgM	79	1.35	0.087	0.0010
Fig4a, Omicron BA.1 vs RF IgG	79	1.23	0.100	0.0005
Fig4b, CS IgG Quant vs ACPA	76	1.15	0.063	0.0141
Fig4b, CS IgG Quant vs RF IgA	86	1.38	0.057	0.0066
Fig4b, CS IgG Quant vs RF IgM	86	1.36	0.059	0.0079
Fig4b, CS IgG Quant vs RF IgG	86	1.28	0.066	0.0046
Fig4b, CS IgM Quant vs ACPA	69	1.26	0.083	0.1740
Fig4b, CS IgM Quant vs RF IgA	75	1.41	0.084	0.1346
Fig4b, CS IgM Quant vs RF IgM	75	1.49	0.083	0.1352
Fig4b, CS IgM Quant vs RF IgG	75	1.34	0.077	0.0967
Fig4b, HSP60 G Quant vs ACPA	83	1.15	0.047	0.0041
Fig4b, HSP60 G Quant vs RF IgA	93	1.34	0.048	0.0224
Fig4b, HSP60 G Quant vs RF IgM	93	1.30	0.054	0.0137
Fig4b, HSP60 G Quant vs RF IgG	93	1.22	0.057	0.0215
Fig4b, HSP60 M Quant vs ACPA	79	1.12	0.140	0.0685

Fig4b, HSP60 M Quant vs RF IgA	89	1.30	0.114	0.0967
Fig4b, HSP60 M Quant vs RF IgM	89	1.27	0.106	0.0333
Fig4b, HSP60 M Quant vs RF IgG	89	1.19	0.089	0.0181
Fig4b, HSP70 G Quant vs ACPA	80	1.13	0.094	0.0728
Fig4b, HSP70 G Quant vs RF IgA	90	1.31	0.078	0.0157
Fig4b, HSP70 G Quant vs RF IgM	90	1.28	0.093	0.0246
Fig4b, HSP70 G Quant vs RF IgG	90	1.20	0.113	0.0213
Fig4b, HSP70 M Quant vs ACPA	82	1.14	0.071	0.0149
Fig4b, HSP70 M Quant vs RF IgA	92	1.33	0.070	0.0172
Fig4b, HSP70 M Quant vs RF IgM	92	1.30	0.069	0.0100
Fig4b, HSP70 M Quant vs RF IgG	92	1.22	0.065	0.0067
Fig5, CS-IgG	112	1.44	0.056	0.0036
Fig5, CS-IgM	100	1.52	0.050	0.1812
Fig5, HSP60-IgG	119	1.41	0.052	0.0062
Fig5, HSP60-IgM	114	1.35	0.098	0.0418
Fig5, HSP70-IgG	116	1.37	0.095	0.0534
Fig5, HSP70-IgM	117	1.39	0.053	0.0077

B. Retrospective minimum detectable effect (MDE), 80% power, two-sided $\alpha = 0.05$

Comparison	n (ref vs comparator)	Residual SD (log scale)	MDE (Cohen's d)	MDE (equivalent fold-change)	Observed fold-change
Fig 2/3 large-group: ACPA-dominant vs NC (QuantiVac)	44 vs 70	1.24	0.54	1.96×	3.14×
Fig 2/3 large-group: ACPA-dominant vs SSA/SSB-dominant (QuantiVac)	44 vs 24	1.24	0.72	2.45×	3.30×
Fig 2/3 large-group: ACPA-dominant vs dsDNA-dominant (QuantiVac)	44 vs 33	1.24	0.65	2.25×	2.58×
Fig 2/3 large-group: ACPA-dominant vs APS (QuantiVac)	44 vs 15	1.24	0.85	2.88×	2.78×
Fig 3 detailed-group: smallest subgroup (aPL+dsDNA) vs	41 vs 7	1.24	1.17	4.28×	n/a (exploratory)

ACPA+RF (QuantiVac)					
Fig 5: smallest nAAb comparison, anti-CS IgM (ACPA-dominant vs NC)	43 vs 57	0.85	0.57	1.63×	1.77×

Table legend. (A) Variance inflation factors (VIF) and Cook's distance were computed for every ordinary least-squares regression model reported in the manuscript (Figures 2–5; log-transformed outcome, HC3-robust standard errors used for inference). A VIF above 5–10 is conventionally considered indicative of problematic multicollinearity; a Cook's distance approaching or exceeding 1 is conventionally considered indicative of a single observation materially influencing the fitted model. Shapiro–Wilk p-values are reported for transparency; departures from normality ($p < 0.05$) motivated the use of HC3-robust standard errors throughout rather than reliance on the normality assumption. (B) Because sample sizes were determined by residual-specimen availability rather than prospective power calculation, retrospective minimum detectable effects (MDE) were calculated post hoc from the achieved group sizes and the observed residual standard deviation (log scale) of the corresponding fitted model, using the standard two-sample t-test power approximation (80% power, two-sided $\alpha = 0.05$). MDE is reported both as a standardized effect size (Cohen's d) and as the equivalent fold-change on the original (untransformed) scale, obtained by exponentiating d × residual SD. Observed fold-changes (from the corresponding adjusted model) are shown for comparison; where the observed effect exceeds the MDE, the analysis was adequately powered to detect it. NC, autoantibody-negative diagnostic samples; ACPA, anti-citrullinated protein antibodies; SD, standard deviation.
